# Detecting the molecular scars of evolution in the *Mycobacterium tuberculosis* complex by analyzing interrupted coding sequences

**DOI:** 10.1186/1471-2148-8-78

**Published:** 2008-03-06

**Authors:** Caroline Deshayes, Emmanuel Perrodou, Daniel Euphrasie, Eric Frapy, Olivier Poch, Pablo Bifani, Odile Lecompte, Jean-Marc Reyrat

**Affiliations:** 1 Université Paris Descartes, Faculté de Médecine René Descartes, Paris Cedex 15, F-75730, France; 2 Inserm, U570, Unité de Pathogénie des Infections Systémiques, Paris Cedex 15, F-75730, France; 3 Laboratoire de Biologie et Génomique Structurales, IGBMC CNRS/INSERM/ULP, BP 163, 67404 Illkirch Cedex, France; 4 Institut Pasteur de Bruxelles, Laboratoire Tuberculose et Mycobactéries, Brussels, Belgium

## Abstract

**Background:**

Computer-assisted analyses have shown that all bacterial genomes contain a small percentage of open reading frames with a frameshift or in-frame stop codon We report here a comparative analysis of these interrupted coding sequences (ICDSs) in six isolates of *M. tuberculosis*, two of *M. bovis *and one of *M. africanum *and question their phenotypic impact and evolutionary significance.

**Results:**

ICDSs were classified as "common to all strains" or "strain-specific". Common ICDSs are believed to result from mutations acquired before the divergence of the species, whereas strain-specific ICDSs were acquired after this divergence. Comparative analyses of these ICDSs therefore define the molecular signature of a particular strain, phylogenetic lineage or species, which may be useful for inferring phenotypic traits such as virulence and molecular relationships. For instance, *in silico *analysis of the W-Beijing lineage of *M. tuberculosis*, an emergent family involved in several outbreaks, is readily distinguishable from other phyla by its smaller number of common ICDSs, including at least one known to be associated with virulence. Our observation was confirmed through the sequencing analysis of ICDSs in a panel of 21 clinical *M. tuberculosis *strains. This analysis further illustrates the divergence of the W-Beijing lineage from other phyla in terms of the number of full-length ORFs not containing a frameshift. We further show that ICDS formation is not associated with the presence of a mutated promoter, and suggest that promoter extinction is not the main cause of pseudogene formation.

**Conclusion:**

The correlation between ICDSs, function and phenotypes could have important evolutionary implications. This study provides population geneticists with a list of targets, which could undergo selective pressure and thus alters relationships between the various lineages of *M. tuberculosis *strains and their host. This approach could be applied to any closely related bacterial strains or species for which several genome sequences are available.

## Background

Recent *in silico *surveys showed that most bacterial genomes contain interrupted coding sequences (ICDSs) [1-3]. These ICDSs generally result from the insertion or deletion of nucleotides, affecting the frame read and splitting the original coding sequence into two or more smaller open reading frames. These mutations may also result in a shift in reading frame, thereby altering the carboxy-terminus of the protein. ICDSs may be present in genes with known or unknown functions, or in hypothetical open reading frames [4]. Reported prokaryotic genomes have a mean of 74 ICDSs per genome, corresponding to 1 to 5% of the genes present, irrespective of genome size or GC content [2,3]. One of the few exceptions is the genome of *M. leprae*, which contains about 30% ICDSs, frequently described as pseudogenes [2,5]. The accumulation of mutations in this species is thought to be due to the loss of the proofreading activity of the DnaQ subunit of DNA polymerase III [6]. A similar sort of reductive evolution is also observed in the case of *M. ulcerans *[7] or for species of the genus *Rickettsiales *[8]. ICDSs may correspond to authentic mutations, generally resulting in a loss of function, but may in some cases reflect sequencing errors. These sequencing errors are misleading when conducting genomic analysis, but have been shown to account for only some of the detected ICDSs [4,9-12]. Most ICDSs correspond to authentic mutations and can therefore be compared between strains, making it possible to explore conserved and unique mutation events.

The availability of complete genomes sequences for genetically related organisms has facilitated comparative analyses of ICDSs. This simple concept, which has not been reported before, enables to investigate evolutionary relationship between isolates or species. In this study, we took the finished genome of two mycobacterial species as a model: *M. tuberculosis*, which causes tuberculosis in humans, and *M. bovis*, which principally causes tuberculosis in ruminants. We also studied six phylogenetically distinct isolates of *M. tuberculosis *– H37Rv, CDC1551, Haarlem, F11, C [13], and 210 (a representative of the W-Beijing family) and *M. africanum*, a species of the *M. tuberculosis *complex for which the genome sequence is still at the assembly step. These isolates are different from each other as they belong to distinct evolutionary branches of the *M. tuberculosis *species, *sensu stricto (s.s)*, yet more closely related to each other than to the more distantly related members of the *M. tuberculosis *complex (*M. africanum*, *M. bovis*, *M. microti *and *M. pinnipedii*) [14]. The W-Beijing family is a clonal group of highly successful *M. tuberculosis *strains associated with multiple outbreaks [15]. This family is one of the oldest lineages to diverge as determined by single nucleotide polymorphism (SNP) and region of deletion analysis [14]. In contrast, H37Rv, the first *M. tuberculosis *strain to be completely sequenced is believed to be one of the most recent (youngest) lineages of *M. tuberculosis *[14,16]. Strain CDC1551 belongs to a lineage that branched between the W-Beijing and the H37Rv isolates. Overall these three isolates represent 3 different genetic groups of the species [14-17]. These isolates have been studied in detail and display differences in genotype [14,18], phenotype and virulence properties [19,20]. By comparing the open reading frames containing frameshifts in these organisms, we showed that ICDSs could be classified as "common to all strains" or "lineage- or strain-specific". The common ICDSs probably correspond to mutations occurring before the divergence of the isolates, whereas lineage- or strain-specific ICDSs correspond to more recently acquired mutations. Thus, ICDS investigation can be used to characterize the molecular scars of evolutionary relationships between organisms and may well provide a unique molecular signature for a particular strain or species, complementary to single nucleotide polymorphism (SNP) and other molecular markers analyses for the characterization of strain variation [18,21]. We also show that ICDS formation is not associated with mutation in the promoter region. The present data suggests that promoter extinction is not a major event in the "pseudogenization" process. To experimentally prove that ICDSs comparison is a powerful phylogenomic tool, we analyzed 21 clinical *M. tuberculosis *isolates for their ICDS content. We showed that the W-Beijing lineage differs from the other TB phyla by a lower number of common ICDSs, confirming early divergence with *M. tuberculosis s.s *strains. ICDS characterization in addition to phylogenetic investigations or typing can be used to select strains or phenotypes for studies of particular phenotypic characters, such as virulence. Indeed, as frameshift acquisition may lead to a loss of function, researchers should consider the possible presence of ICDS before choosing a strain or species for investigating a particular phenotype.

## Results

### Detecting the molecular scars of evolution in *M. tuberculosis *and in *M. bovis*

**Figure 1:**
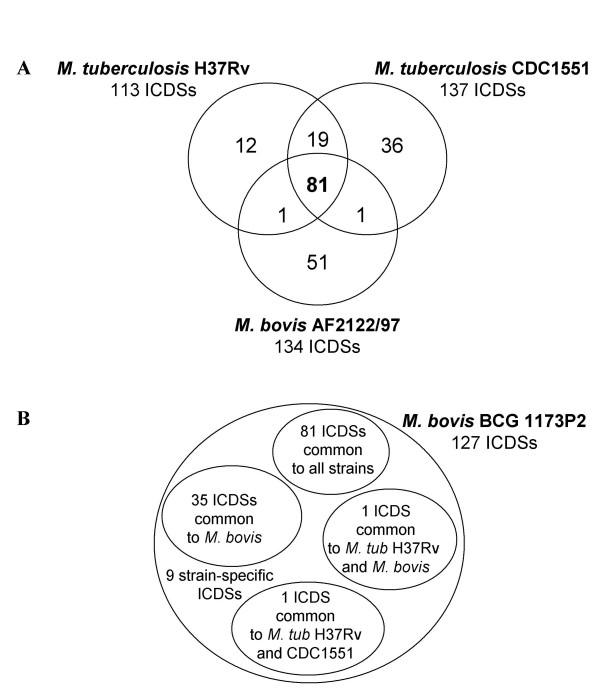
**A- **Schematic representation of the ICDSs common to *M. tuberculosis *H37Rv, CDC1551 and *M. bovis *AF2122/97 or specific to one of these strains. The total number of ICDSs is indicated. **B- **Schematic representation of the ICDSs of *M. bovis *BCG 1173P2 compared to the other analyzed strains.

Comparative analyses of frameshift-containing genes require the complete genome sequences of closely related organisms. The TB complex, which includes two recently sequenced species and at least 6 accessible strains, is therefore a highly suitable model. We investigated ICDSs in *M. tuberculosis *and in *M. bovis*. The genome sequence of *M. tuberculosis *H37Rv has been available since 1998 and has recently been re-annotated [22,23]. The genome sequences of *M. tuberculosis *strain CDC1551 and *M. bovis *have been characterized independently [18,24]. The great advantage of studying this model system is that the evolution of these two species and the phylogenetic links between them are well documented [25]. The *M. tuberculosis *genomes (CDC1551 and H37Rv) have nucleotide sequences more than 99.95 % identical to that of *M. bovis *[18,24]. The three genomes were screened for the presence of ICDSs. To this end, the genomic sequences of each predicted ICDS [3] were extracted for each strain or species and compared between them. Each common or specific ICDS was then analyzed manually to characterize the molecular event leading to the detected frameshift. The genome of H37RV contains 113 ICDSs, whereas CDC1551 has 137 ICDSs and *M. bovis *has 134 ICDSs, corresponding to about 2% of the total coding sequences [3]. These organisms have similar numbers of ICDSs, but the alterations do not always affect the same genes. We therefore investigated whether some of these ICDSs were common to all three organisms. We compared the nucleotide and deduced amino-acid sequences of each frameshift-containing open reading frame in the three organisms. We found that 81 of the frameshift-containing genes were common to all three strains (Figure 1A, Table 1), and were identical at the molecular level. The proteins affected by these frameshifts included proteins of unknown function as well as annotated and/or characterized proteins (Table 1). The fact that these three mycobacterial genomes were sequenced and assembled independently suggests that these 81 common ICDSs correspond to authentic frameshift-containing genes rather than sequencing errors. These results indicate that these 81 ICDSs correspond to frameshifts acquired before the splitting of the *M. tuberculosis *and *M. bovis *species (Table 1). Alternatively, the same 81 genetic mutations may result from convergent evolution and hence have occurred independently in all three genomes, a highly unlikely scenario.

**Table 1:** List of the 81 ICDSs common to *M. tuberculosis *H37Rv, CDC1551, *M. bovis *AF2122/97 and *M. africanum *GM041182.

*M. tub *H37Rv	*M. tub *CDC1551	*M. bov *AF2122/97	*M. bov *BCG 1173P2	*M. africanum*	Putative function	Functional classification
0002 (Rv0151c *588 aa*)	0004	0006	0109	ICDS	PE family protein	PE/PPE
0003 (Rv0152c *525 aa*)	0005	0007	0004	ICDS	PE family protein	PE/PPE
0007 (Rv0366c *197 aa *– Rv0367c *129 aa*)	0011	0010	0110	ICDS	Conserved hypothetical	Unknown
0009 (Rv0393 *441 aa*)	0012	0011	0008	ICDS	Conserved hypothetical	Unknown
0010 (Rv0520 *116 aa *– Rv0521 *101 aa*)	0014	0013	0012	ICDS	Dimethylglycine N-methyltransferase	Intermediary metabolism
0012 (Rv0601c *157 aa*)	0017	0018	0016	ICDS°	Two-component sensor kinase	Regulation
0014 (Rv0635 *158 aa *– Rv0636 *142 aa*)	0019	0020	0111	ICDS	Conserved hypothetical	Unknown
0015 (Rv0636 *142 aa *– Rv0637 *166 aa*)	0020	0021	0112	ICDS	Conserved hypothetical	Unknown
0017 (Rv0724A *112 aa *– Rv0725c *301 aa*)	0022	0023	0020	ICDS	Conserved hypothetical	Unknown
0020 (Rv0865 *160 aa*)	0026	0085	0113	ICDS	Molybdopterin biosynthesis protein	Intermediary metabolism
0021 (Rv0890c *882 aa *– Rv0891c *285 aa*)	0027	0025	0114	ICDS	Transcriptional regulator	Regulation
0023 (Rv1034c *130 aa *– Rv1035c *228 aa*)	0030	0030	0031	ICDS	Transposase	IS/phage
0024 (Rv1035c *228 aa *– Rv1036c *112 aa*)	0031	0031	0032	ICDS	Transposase	IS/phage
0025 (Rv1041c *287 aa *– Rv1042c *135 aa*)	0032	0086	0034	ICDS	Transposase	IS/phage
0026 (Rv1104 *229 aa*)	0034	0087	0115	ICDS	Esterase	Intermediary metabolism
0027 (Rv1104 *229 aa*)	0035	0088	0037	ICDS	Esterase	Intermediary metabolism
0028 (Rv1105 *171 aa*)	0036	0033	0036	ICDS	Para-nitrobenzyl esterase	Intermediary metabolism
0029 (Rv1119c *49 aa *- Rv1120c* 164 aa*)	0037	0034	0039	ICDS	Conserved hypothetical	Unknown
0030 (Rv1136 *113 aa*)	0039	0089	0040	ICDS	Enoyl-CoA	Lipid metabolism
0032 (Rv1149 *135 aa *– Rv1150 *183 aa*)	0041	0090	0041	ICDS	Transposase	IS/phage
0033 (Rv1163 *201 aa *– Rv1164 *246 aa*)	0042	0035	0116	ICDS	Nitrate reductase NarI-J	Intermediary metabolism
0035 (Rv1203c *194 aa *– Rv1204c *562 aa*)	0044	0036	0043	ICDS	Conserved hypothetical	Unknown
0036 (Rv1413 *171 a*)	0046	0041	0047	ICDS	Conserved hypothetical	Unknown
0040 (Rv1662 *1602 aa *– Rv1663 *502 aa*)	0053	0043	0117	ICDS	Polyketide synthase Pks8/17	Lipid metabolism
0041 (Rv1687c *255 aa*)	0054	0132	0128	ICDS°	ATP binding protein, ABC transporter	Cell wall, process
0043 (Rv1735c *166 aa*)	0056	0112	0050	ICDS	Malic acid transport protein	Cell wall, process
0046 (Rv1878 *450 aa*)	0061	0052	0118	ICDS°	Glutamine synthetase GlnA3	Intermediary metabolism
0047 (Rv1888A *58 aa *– Rv1889c *118 aa*)	0062	0053	0119	ICDS	Conserved hypothetical	Unknown
0048 (Rv1931c *259 aa*)	0064	0054	0058	ICDS	Conserved hypothetical	Unknown
0049 (Rv1949c *319 aa *– Rv1950c *63 aa*)	0065	0055	0059	ICDS	Conserved hypothetical	Unknown
0050 (Rv2013 *159 aa *– Rv2014 *196 aa*)	0066	0056	0061	ICDS°	Transposase	IS/phage
0051 (Rv2086 *201 aa*)	0067	0093	0062	ICDS	Conserved hypothetical	Unknown
0052 (Rv2086 *201 aa *– Rv2087 *76 aa*)	0068	0058	0063	ICDS	Conserved hypothetical	Unknown
0053 (Rv2087 *76 aa*)	0069	0094	0064	ICDS	Conserved hypothetical	Unknown
0054 (Rv2095c *316 aa *– Rv2096 *332 aa*)	0070	0133	0129	ICDS	Conserved hypothetical	Unknown
0058 (Rv2321 *182 aa *– Rv2322c *221 aa*)	0074	0060	0067	ICDS	Ornithine aminotransferase RocD1	Intermediary metabolism
0059 (Rv2325 *282 aa *– Rv2326c *697 aa*)	0075	0096	0120	ICDS	Conserved hypothetical	Unknown
0060 (Rv2331 *129 aa*)	0076	0134	0130	ICDS	Hypothetical	Unknown
0061 (Rv2337 *372 aa *– Rv2338c *318 aa*)	0077	0097	0068	ICDS	Conserved hypothetical	Unknown
0062	0081	0065	0076	ICDS	Hydrogenase nickel incorporation protein HypB	Intermediary metabolism
0063 (Rv2877c *287 aa *– Rv2878c *173 aa*)	0082	0099	0077	ICDS°	Conserved hypothetical	Unknown
0065 (Rv2943A *177 aa *– Rv2944 *239 aa*)	0083	0066	0079	ICDS	Transposase	IS/phage
0068 (Rv3128c *338 aa*)	0088	0068	0082	ICDS	Conserved hypothetical	Unknown
0069 (Rv3152 *410 aa *– Rv3153 *211 aa*)	0089	0100	0121	ICDS	NADH dehydrogenase I	Intermediary metabolism
0070 (Rv3172c *160 aa*)	0090	0069	0083	ICDS	Conserved hypothetical	Unknown
0071 (Rv3200c *355 aa*)	0091	0101	0122	ICDS	Hypothetical	Unknown
0075 (Rv3349c *246 aa*)	0100	0102	0085	ICDS°	Transposase	IS/phage
0076 (Rv3351c *264 aa *– Rv3352c *123 aa*)	0101	0071	0088	ICDS	Oxidoreductase	Intermediary metabolism
0077 (Rv3352c *123 aa *– Rv3353c *86 aa*)	0102	0072	0089	ICDS	Oxidoreductase	Intermediary metabolism
0079 (Rv3419c *344 aa*)	0104	0135	0131	ICDS	O-sialoglycoprotein endopeptidase	Intermediary metabolism
0080 (Rv3420c *158 aa *– Rv3421c *211 aa*)	0105	0104	0092	ICDS°	Conserved hypothetical	Unknown
0083	0108	0107	0095	ICDS	Transposase	IS/phage
0084 (Rv3636 *115 aa *– Rv3637 *166 aa*)	0111	0076	0097	ICDS	Transposase	IS/phage
0087 (Rv3741c *224 aa *– Rv3742c *131 aa*)	0114	0078	0099	ICDS°	Aromatic-ring hydroxylase	Intermediary metabolism
0088 (Rv3770A *61 aa *– Rv3770B *64 aa*)	0115	0079	0100	ICDS°	Transposase	IS/phage
0089 (Rv 3844 *164 aa *– Rv3845 *120 aa*)	0116	0136	0132	ICDS	Transposase	IS/phage
0090 (Rv3866 *283 aa *– Rv3867 *183 aa*)	0117	0109	0123	ICDS	Conserved hypothetical	Unknown
0091 (Rv3880c *115 aa *– Rv3881 *460 aa*)	0118	0137	0133	ICDS	Conserved hypothetical	Unknown
0095 (Rv3900c *311 aa*)	0122	0083	0124	ICDS	Conserved hypothetical	Unknown
0097 (Rv3913 *335 aa *– Rv3914 *116 aa*)	0123	0111	0001	ICDS	Thioredoxin reductase	Intermediary metabolism
0098	0124	0032	0035	ICDS	Conserved hypothetical	Unknown
0099 (Rv3386 *234 aa *– Rv3387 *225 aa*)	0125	0103	0090	ICDS	Transposase	IS/phage
0100 (Rv0342 *640 aa *– Rv0343 *493 aa*)	0126	0009	0125	ICDS	Isoniazid inductible gene protein	Cell wall, process
0101 (Rv0763c *69 aa *– Rv0764c *451 aa*)	0127	0084	0126	ICDS	Cytochrome P450	Intermediary metabolism
0102 (Rv1858 *264 aa *– Rv1859 *369 aa*)	0128	0092	0127	ICDS	Molybdenum transport ABC transporter	Cell wall, process
0103 (Rv0449c *439 aa*)	0129	0113	0010	ICDS	Conserved hypothetical	Unknown
0104 (Rv0471c *162 aa*)	0130	0114	0011	ICDS	Hypothetical	Unknown
0105 (Rv0859 *403 aa *– Rv0860 *720 aa*)	0131	0115	0024	ICDS°	Acyl-CoA thiolase FadA and dehydrogenase FadB	Lipid metabolism
0106 (Rv0880 *143 aa *– Rv0881 *288 aa*)	0132	0116	0025	ICDS°	Transcriptional regulator	Information pathway
0107 (Rv0997 *143 aa*)	0133	0117	0029	ICDS	Hypothetical	Unknown
0108 (Rv1041c *287 aa *– Rv1042c *135 aa*)	0134	0118	0033	ICDS°	Transposase	IS/phage
0109 (Rv1104 *229 aa *– Rv1105 *171 aa*)	0135	0119	0038	ICDS	Para-nitrobenzyl esterase	Intermediary metabolism
0110 (Rv1221 *257 aa *– Rv1222 *154 aa*)	0136	0120	0044	ICDS	Alternative sigma factor SigE	Information pathway
0111 (Rv1752c *149 aa*)	0137	0121	0051	ICDS	Conserved hypothetical	Unknown
0112 (Rv1961 *164 aa*)	0138	0122	0060	ICDS	Hypothetical	Unknown
0113 (Rv2309c *151 aa*)	0139	0123	0066	ICDS	Integrase	Information pathway
0114 (Rv2420c *127 aa *– Rv2421c *211 aa*)	0140	0124	0070	ICDS	nicotinate-nucleotide adenylyltransferase NadD	Intermediary metabolism
0115 (Rv2732c *205 aa *– Rv2733c *512 aa*)	0141	0125	0073	ICDS	Conserved hypothetical	Unknown
0116 (Rv2922A *94 aa *– Rv2923c *137 aa*)	0142	0126	0078	ICDS	Acylphosphatase AcyP	Intermediary metabolism
0117 (Rv3774 *274 aa *– Rv3775 *274 aa*)	0143	0127	0101	ICDS°	Enoyl-CoA hydratase EchA21 and lipase LipE	Lipid metabolism
0119 (Rv2599 *143 aa *– Rv2600 *133 aa*)	0144	0098	0134	ICDS°	Conserved hypothetical	Unknown

ICDS number (variable, according to the strain), the size of the predicted protein and its putative function are indicated. The corresponding ORF numbers in *M. tuberculosis *H37Rv are indicated in brackets. "°" indicates ICDSs containing additional mutations with respect to *M. tuberculosis *H37Rv, CDC1551 and *M. bovis *AF2122/97.

The two *M. tuberculosis s.s *strains were found to have 19 additional common ICDSs, raising their total number to 100 (Figure 1A, Table 2). This suggests that the 19 additional mutations common to these two strains but not to *M. bovis *were acquired post-divergence of *M. tuberculosis *and *M. bovis*. One ICDS in *M. bovis *(ICDS0046, Mb1789c-Mb1790c) was present in *M. tuberculosis *CDC1551 (ICDS0057, MT1807) but not in *M. tuberculosis *H37Rv (Rv1759c). This mutation (deletion of one G) was identical in the *M. bovis *and *M. tuberculosis *CDC1551 strains, but an additional mutation was present close to this mutation in the *M. bovis *genome. One ICDS in *M. bovis *(ICDS0128, Mb3813-Mb3814) was also present in *M. tuberculosis *H37Rv (ICDS0118, Rv3784-Rv3785) but not in *M. tuberculosis *CD1551 (MT3893) (Table 2).

**Table 2:** 

*M. tub *H37Rv	*M. tub *CDC1551	M. bovis AF2122/97	*M. tub *210	*M. africanum*	Putative function	Functional classification
0001 (Rv0095c *136 aa*)	0003	Mb0098c *260 aa*	ICDS	Not Found	Conserved hypothetical	Unknown
0005 (Rv0325 *74 aa *– Rv0326 *151 aa*)	0010	Mb0333 *229 aa*	FL	FL	Hypothetical	Unknown
0011 (Rv0590 *275 aa *– Rv0590A 9 0 *84 aa*)	0016	Mb0605 *343 aa*	FL	FL	MCE-family protein	Virulence, detox, adapt
0013 (Rv0618 *231 aa *– Rv0619 1 8 *181 aa*)	0018	Mb0635 *394 aa*	ICDS	FL	Galactose-1-phosphate uridylyltransferase	Intermediary metabolism
0022 (Rv0924c *428 aa *– Rv0925c *245 aa*)	0028	Mb0948c *684 aa*	ICDS	ICDS	Manganese transport protein MntH	Cell wall, process
0031 (Rv1145 *303 aa *– Rv1146 *470 aa*)	0040	Mb1177 *781 aa*	FL	FL	Transmembrane transport protein MmpL13	Cell wall, process
0037 (Rv1503c *182 aa *– Rv1504c *199 aa*)	0048	Mb1542c *382 aa*	ICDS	FL	Conserved hypothetical	Unknown
0038 (Rv1549 *175 aa *– Rv1550 *571 aa*)	0051	Mb1576 *647 aa*	ICDS	FL	Fatty-acid-coA ligase FadD11	Lipid metabolism
0039 (Rv1553-Rv1554 *247 aa – 126 aa*)	0052	Mb1579 *374 aa*	ICDS°	ICDS°	Fumarate reductase	Intermediary metabolism
0045 (Rv1792 *59 aa*)	0058	Mb1820 *98 aa*	ICDS	FL	ESAT-6-like protein EsxM	Cell wall, process
0055 (Rv2227 *233 aa*)	0072	Mb2252 *124 aa*	ICDS	FL	Conserved hypothetical	Unknown
0066 (Rv2946c *1616 aa *– Rv2947 *496 aa*)	0084	Mb2971c *2112 aa*	FL	FL	Polyketide synthase Pks15/1	Lipid metabolism
0067 (Rv2974c *470 aa *– Rv2975c *84 aa*)	0085	Mb2999c *553 aa*	FL	FL	Conserved hypothetical	Unknown
0072 (Rv3233c *196 aa *– Rv3234c *271 aa*)	0092	Mb3262c *469 aa*	FL	FL	Conserved hypothetical	Unknown
0073 (Rv3337 *128 aa *– Rv3338 *214 aa*)	0094	Mb3370 *297 aa*	ICDS	FL	Conserved hypothetical	Unknown
0078 (Rv3373 *213 aa *– Rv3374 *82 aa*)	0103	Mb3408 *296 aa*	ICDS	FL	Enoyl-CoA hydratase EchA18	Lipid metabolism
0085 (Rv3725 *309 aa*)	0112	Mb3752 *333 aa*	FL	FL	Oxidoreductase	Intermediary metabolism
0086 (Rv3738c *315 aa *– Rv3739c *77 aa*)	0113	Not determined	ICDS	ICDS	PPE family protein	PE/PPE
0094 (Rv3897c *210 aa *– Rv3898c *110 aa*)	0121	Mb3927c *329 aa*	FL	FL	Conserved hypothetical	Unknown

***M. tub *H37Rv**	**M. bovis AF2122/97**	***M. tub *CDC1551**	***M. tub *210**	***M. africanum***	**Putative function**	**Functional classification**

0118 (Rv3784 *326 aa *– Rv3785 *357 aa*)	0128	MT3893 *712 aa*	NT	ICDS	NAD-dependent epimerase/dehydratase	Information pathway

***M. tub *CDC1551**	**M. bovis AF2122/97**	***M. tub *H37Rv**	***M. tub *210**	***M. africanum***	**Putative function**	**Functional classification**

0057 (MT1806 *820 aa *– MT1807 *94 aa*)	0046	Rv1759c *914 aa*	NT	NT	PE_PGRS family protein	PE/PPE

List of the 19 ICDSs common to *M. tuberculosis *H37Rv and CDC1551, the ICDSs common to *M. tuberculosis *H37Rv and *M. bovis *AF2122/97 and the ICDSs common to *M. tuberculosis *CDC1551 and *M. bovis *AF2122/97. ICDS number (variable, according to strain), the size of the predicted protein and its putative function are indicated. The genes that do not contain a frameshift in either *M. tuberculosis *strain 210 and in *M. africanum *and that correspond to a full-length ORF are noted "FL". "NT", not tested.

The availability of genomic resources for *M. tuberculosis *is increasing exponentially. This enabled us to investigate the presence or absence of these shared ICDSs in the Haarlem, F11, and C strains, the genomic sequences of which are currently at the assembly stage at the Broad Institute [26]. As the sequence of these genomes is in progress, the total number of frameshift-containing genes in these genomes cannot yet be accurately determined; nonetheless, it is possible to check whether the 81 ICDSs present in *M. bovis *and in other *M. tuberculosis *strains are present in these strains. All 81 ICDSs common to all three strains previously tested were also present in Haarlem and F11 strains, while 79 were present in the C strain (corresponding H37Rv ORFs ICDS0103 and ICDS0105 were full-length in this strain) (see Additional file 1). Noteworthy, was the identification of additional mutations in the vicinity (≤ 200 bp) of the original frameshift (see additional file 1). We next investigated whether the 19 ICDSs common to all *M. tuberculosis s.s *strains were present in the other clinical isolates. In each case, the ICDSs were also present in the three strains (Haarlem, F11, and C), but accompanied, in some cases, by additional mutations in the flanking region (see Additional file 1). Thus, 98 frameshift-containing genes were found to be conserved in all five *M. tuberculosis *strains analyzed.

The recently published *M. bovis *BCG genome sequence is of a particular interest in this respect [27]. This strain, which is currently used for vaccination in humans, was derived from *M. bovis *after 13 years of repetitive passages *in vitro *[28]. A number of genetic differences, such as deletions and duplications had already been identified in the BCG strain [29,30], but large amounts of additional information have now been obtained from its genome sequence. According to our investigation, *M. bovis *BCG 1173P2 contains 127 ICDSs in total, 9 of which are strain-specific (Figure 1B). The 81 ICDSs common to the 3 other isolates are also present in this strain (Table 1) and 35 ICDSs are common to the *M. bovis *strain. We detected frameshift-containing genes in *M. bovis *AF2122/97 that corresponded to full-length ORFs in *M. bovis *BCG 1173P2, suggesting that this *M. bovis *strain is not the direct progenitor of the BCG vaccine (see Additional file 2).

### Strain-specific ICDSs reflect newly acquired mutations and are a useful phylogenetic tool

Eighty-one ICDSs were common to all three strains, but some were specific to one strain only: 12 for *M. tuberculosis *H37Rv (see Additional file 3), 36 for CDC1551 (see Additional file 4) and 51 for *M. bovis *(see Additional file 2, Figure 1A). The proportion of ICDSs that were strain-specific was highly variable. These ICDSs accounted for 10% of all ICDSs in H37Rv, 26% in CDC1551 and 38% in *M. bovis*. The much larger proportion of strain-specific ICDSs in CDC1551 than in H37Rv strain is surprising, and we currently have no reasonable explanation for this phenomenon. A plausible hypothesis is that the genome sequence of CDC1551 strain has not been re-sequenced like the H37Rv genome sequence [22,28]. Strain-specific frameshift-containing genes most likely correspond to mutations acquired after the divergence of these strains. Like the common ICDSs, these events affected genes from several classes, including "unknown or hypothetical ORFs", "intermediary metabolism" and "cell wall, process" (Additional files 2, 3 and 4). As stated above, few of these strain-specific ICDSs may correspond to errors introduced during the sequencing procedure [4,11], but such errors would nonetheless have only a slight effect on the overall outcome of the comparative analysis.

This study shows that the genome sequence of *M. tuberculosis *contains ICDSs that have been acquired during the evolution of this species. The pool of ICDSs can be classified into ICDSs common to a set of strains or species and ICDSs specific to a particular strain-lineage or strain, revealing genetic differences between strains or species.

### Using ICDS comparisons to type W-Beijing strains and other *M. tuberculosis *lineages

W-Beijing is a lineage of *M. tuberculosis *that has attracted considerable attention. Indeed, strains of this lineage have been implicated in severe outbreaks and have been shown to have different genetic and phenotypic properties [20,21,31]. The genome of a strain of the W-Beijing family (strain 210) is currently sequenced but not yet fully assembled; nevertheless it can be consulted in homology searches. Consequently the total number of frameshift-containing genes in this species and the full characterization of specific ICDSs remain elusive. It is however possible to screen for the presence of ICDSs in this strain.

We first investigated whether the 81 frameshift-containing genes common to all strains were also present in the genome of strain 210. All 81 of these genes also contained the same frameshift in strain 210, in agreement with the data described above. This suggests that these 81 frameshift mutations were acquired before the divergence of strain 210 from these other strains. We then investigated the 19 genes containing frameshifts common to the five strains of *M. tuberculosis *(H37Rv, CDC1551, Haarlem, F11, C) but not to *M. bovis*. We found that eight of these 19 genes contain no frameshift in strain 210, and hence corresponded to full-length ORFs (Table 2). Three genes contained frameshifts corresponding to those observed in strains CDC1551, H37Rv, Haarlem, F11 and C, but also contained additional mutations in the corresponding flanks (≤ 200 bp) of the original frameshift (Table 2). The remaining 11 ICDSs corresponded to frameshift-containing genes common to all six TB strains examined (CDC1551, H37Rv, Haarlem, F11, C, 210) and the events were identical at the molecular level. Thus, the 19 frameshift-containing genes in the two TB strains (CDC1551 and H37Rv) displayed polymorphism in strain 210 and 11 of these identified ICDSs were common to all six TB strains examined. Some of these ICDSs display no further mutation (the gene contains the frameshift alone), whereas others have acquired additional mutations, contributing to the "pseudogenization" process (data not shown).

We then investigated the eight ICDSs showing polymorphism in *M. tuberculosis *in 21 strains of the W-Beijing lineage from several phylogenetic groups (Table 3). The eight loci were amplified by PCR, sequenced and the nucleotide sequence was compared with that of strains 210 and H37Rv. In all W-Beijing strains tested, the eight genes were full-length, with sequences 100% identical to that in strain 210, excepted for the ICDS0085 where a non-disruptive SNP is present in the region. The W-Beijing lineage is therefore a genetically homogeneous group with fewer ICDSs in common with other TB strains.

**Table 3:** Analysis in 21 W-Beijing isolates of the 8 ICDSs of H37Rv strain corresponding to full-length ORFs in W-Beijing strain 210.

	Finger print	Tracking Number	ICDS 0005	ICDS 0011	ICDS 0031	ICDS 0066	ICDS 0067	ICDS 0072	ICDS 0085	ICDS 0094
**W-Beijing**	**W**	**10648**	FL	FL	FL	FL	FL	FL	NT	FL
	**W**	**565**	FL	FL	FL	FL	FL	FL	FL*	FL
	**W4**	**10775**	FL	FL	FL	FL	FL	FL	FL*	FL
	**W14**	**3617**	FL	FL	FL	FL	FL	FL	FL*	FL
	**W26**	**10270**	FL	FL	FL	FL	FL	FL	FL*	FL
	**W69**	**5418**	FL	FL	FL	FL	FL	FL	FL*	FL
	**W88**	**7052**	FL	FL	FL	FL	FL	FL	FL*	FL
	**W130**	**6707**	FL	FL	FL	FL	FL	FL	FL*	FL
	**W148**	**8561**	FL	FL	FL	FL	FL	FL*	FL*	FL
	**W183**	**7657**	FL	FL	FL	FL	FL	FL	NT	FL
	**W215**	**8963**	FL	FL	FL	FL	FL	FL	FL*	FL
	**W342**	**10644**	FL	FL	FL	FL	FL	FL	FL*	FL

**Ancestral W-Beijing**	**N17**	**3046**	FL	FL	FL	FL	FL	FL	FL*	FL
	**LB**	**8128**	FL	FL	FL	FL	FL	FL	FL*	FL
	**AR**	**12360**	FL	FL	FL	FL	FL	FL	FL*	FL
	**AM**	**4948**	FL	FL	FL	FL	FL	FL	FL*	FL
	**CK**	**6595**	FL	FL	FL	FL	FL	FL	FL*	FL
	**CN1**	**16116**	FL	FL	FL	FL	FL	FL	FL*	FL
	**HE7**	**13454**	FL	FL	FL	FL	FL	FL	FL*	FL
	**HI**	**5116**	FL	FL	FL	FL	FL	FL	FL*	FL
	**KY**	**10583**	FL	FL	NT	FL	FL	FL	FL*	FL

**AF/H37 lineage**	**H37Rv**	**ATCC25618**	ICDS	ICDS	ICDS	ICDS	ICDS	ICDS	ICDS	ICDS

***M. bovis *AF2122/97**			FL	FL	FL	FL	FL	FL	FL	FL

*M. tuberculosis *isolates from various lineages for which chromosomal DNA was used as a template for PCR amplification of the selected locus. "FL" indicates the presence of a full-length ORF identical to that in the *M. tuberculosis *210 strain, "*" indicates an additional mutation acquired in these isolates with respect to *M. tuberculosis *H37Rv, "NT", not tested.

To extend our analysis, we investigate the *M. africanum *strain, which is currently sequenced at the Sanger centre. Similarly to *M. tuberculosis *210 strain, the *M. africanum *genome is still at the assembly step, but can be nevertheless consulted on line. We investigated whether the 81 frameshift containing genes common to all strains tested were also present in the *M. africanum *strain (Table 1). All 81 of these genes also contained a frameshift in *M. africanum*, which suggests that these mutations were acquired before the divergence of the *M. tuberculosis *complex. We then investigated the 19 genes containing frameshift common to the 5 *M. tuberculosis *strains (CDC1551, H37Rv, Haarlem, F11, C). We found that 15 out of these 19 genes were deprived of the frameshift in *M. africanum *and corresponded to full-length ORFs in this strain (Table 2). Eight out of these 15 genes match the wild-type ORFs identified in *M. tuberculosis *strain 210 and other strains of the W-Beijing lineage. In conclusion, the genome of *M. africanum *contains fewer ICDSs in common with the other TB isolates (CDC1551, H37Rv, Haarlem, F11, C) than with the W-Beijing strain and seems genetically closer to this lineage.

### ICDS formation is not correlated with mutation in the promoter region

It has been suggested that pseudogene formation is associated with mutations in the upstream untranslated region, abolishing pseudogene expression to prevent a loss of metabolic function [32]. Once turned off, the gene continues to accumulate mutations, leading to complete pseudogene formation. ICDSs are not pseudogenes in the strict sense of the word. Indeed, the ORF is split into only two or three unframed fragments and can, in theory, revert to a wild-type allele. ICDSs are therefore considered to be ORFs undergoing "pseudogenization" rather than pseudogenes *per se*. Strain-specific ICDSs are, by definition, genes that are mutated in one strain, but not in another. We therefore investigated whether ICDS formation was correlated with mutation in the promoter region. All the intergenic regions (99) located upstream from strain-specific ICDSs of *M. tuberculosis *H37Rv, CDC1551 and *M. bovis *were compared with the corresponding region in the two strains having a wild-type gene. We used as a control the promoter region of randomly selected genes that are full-length in these 3 strains. We compared the level of differences observed in the promoter regions of genes full-length or containing frameshift. Nucleotide differences were observed in 27% of the upstream region of genes containing frameshift (see Additional file 5A), while 20% was observed in the case of the full-length genes (see Additional file 5B), which is not statistically significant using the chi square test. In all but 6 cases for ICDS and 2 cases for full-length genes, the difference in the upstream region was limited to one or two SNPs.

We therefore conclude that ICDS formation is not correlated with mutation in the untranslated upstream region and suggest that either promoter mutations do not play a major role in pseudogene formation in the *M. tuberculosis *complex or that "pseudogenization" is recent.

## Discussion

The presence of frameshift-containing genes in bacterial genomes is well documented [1-3,33]. A few species can bypass such frameshifts, but most do not, generally resulting in a loss of function.

**Figure 2:**
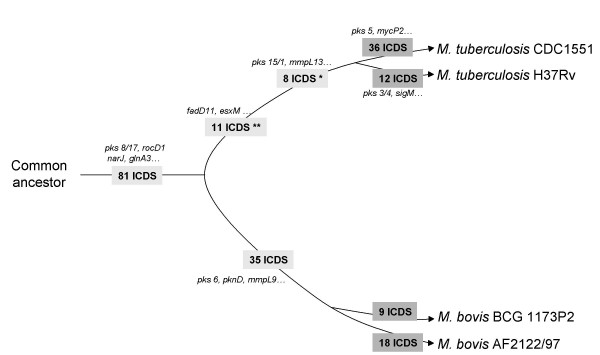
Hypothetical phylogenetic links assessed by comparative analyses of ICDSs. In this schematic representation, the common ancestor gave rise to several branches of strains of the TB complex. Eighty-one frameshifts were acquired during the common evolution of *M. bovis *and *M. tuberculosis*. Since the separation of these species, *M. bovis *has acquired 51 frameshifts, while the branch leading to *M. tuberculosis *isolates has acquired 19 new frameshifts. Since separation of the isolates, *M. tuberculosis *H37Rv has acquired 12 new frameshifts and CDC1551 36 new frameshifts. Common and unique ICDSs are shown in dark and light gray, respectively. "*" these 8 ICDSs correspond to full-length ORF in *M. tuberculosis *210 and in *M. africanum *GM041182. "**" 7 out of these 11 ICDSs correspond to full-length ORF in *M. africanum *GM041182 (Table 2).

We show here that ICDSs can be classified as "common to all strains" or "strain-specific". The ICDSs common to all strains probably correspond to mutations acquired before the divergence of the strains, whereas strain-specific ICDSs correspond to those acquired subsequently (Figure 2). Mutations acquired after the speciation of *M. tuberculosis *from *M. bovis *were also detected. We identified 19 ICDSs common to the five *M. tuberculosis *strains (H37Rv, CDC1551, Haarlem, F11 and C) but not to *M. bovis*, about one-fifth of ICDSs common to all strains. Comparative analyses of ICDSs help to characterize the phylogenetic relationships between highly related strains and species (Figure 2) and could be applied to any bacterial species for which several genome sequences are available. In few cases, ICDSs may correspond to fusion/fission of orthologous genes in other genomes. The detection of this kind of events is due to the method of identification of ICDS but remains however a minor inconvenience [3]. It is however possible that a low percentage of specific ICDSs does correspond to sequencing errors, inducing thus artifactual phylogenetic relationships. Researchers should resequence these regions before assuming that the ICDS corresponds to a frameshift acquisition. Several studies have compared the genome sequences of *M. tuberculosis *CDC1551 and H37Rv, using high-resolution genomics techniques [18]. This has led to the definition of regions containing large-sequence polymorphisms (LSPs, greater than 10 bp) and single nucleotide polymorphisms (SNPs). The SNPs have been investigated in more detail in various clinical isolates, to draw up a global phylogeny of *M. tuberculosis *[17]. Other molecular methods, such as analyses of the deleted regions (deligotyping), variable numbers of tandem repeats (VNTR), mycobacterial interspersed repetitive unit (MIRU) and spoligotyping, have helped to unravel global genomic sequence diversity in this species [34-36]. These techniques are highly useful for epidemiological studies, but as far provide little information pertaining to genetic differences in terms of putative function. In contrast, studies of regions of deletion (RD) have proved useful for both global phylogeny and study of a loss of phenotype in both *M. tuberculosis *and in *M. ulcerans *[25,30,37].

Frameshift acquisition generally leads to a loss of function, as shown in a number of published studies. Loss-of-function associated with the presence of a frameshift has been reported in both *M. tuberculosis *and *M. bovis*. For instance, ICDS0066 in *M. tuberculosis *H37Rv corresponds to a frameshift-containing gene encoding a polyketide synthase (*pks1*). This *pks1 *gene also contains a frameshift in *M. tuberculosis *CDC1551, resulting in two different ORFs: *pks1 *and *pks15*. In contrast, *M. bovis *and *M. leprae *carry a full-length functional *pks1 *gene [38]. The *pks15/1 *gene is now frequently used as a marker in epidemiological studies [39,40] and, interestingly, the *pks *gene contains no frameshift in the W-Beijing strains of *M. tuberculosis *[40], resulting in phenolglycolipid production in most cases [41]. Our analysis shows that the *pks *gene of *M. africanum *is also full-length suggesting that this species produces PGL. This observation suggests that these early strains are more closely related to *M. bovis *or to the last ancestor than other *M. tuberculosis *strains. Similarly, ICDS0067 in *M. bovis *corresponds to a putative frameshift-containing glycosyltransferase gene. The ortholog of this gene has no frameshift in the two strains of *M. tuberculosis *(Rv2958c and MT3034). Functional complementation of *M. bovis *BCG with the Rv2958c gene from *M. tuberculosis *leads to the accumulation of a new metabolite, the diglycosylated phenolglycolipid [42]. Some frameshift-containing genes have been studied experimentally in *M. tuberculosis*, without considering the possibility that these ORFs may well contain frameshift [43,44]. Mutation by homologous recombination has been achieved at the *mntH *and *mmpL13 *loci. In both cases, no detectable phenotype was associated with the mutation. Our data indicate that MmpL13 function should be investigated in a W-Beijing strain or in *M. africanum*. Another example that has not yet been studied is the *pks3 *and *pks4 *genes of *M. tuberculosis *H37Rv, which constitute a single ORF in CDC1551 and in *M. bovis*. This suggests that – like the *pks1 *and *pks15 *genes, which are pseudogenes in *M. tuberculosis *– the *pks3 *and *pks4 *genes are probably not functional in the H37Rv strain. It would therefore be pointless to investigate function in the H37Rv strain by creating mutants in *pks3 *and *pks4 *genes or by expressing constructs encoding the corresponding polypeptides. These examples from previous publications illustrate the major biological impact of frameshift acquisition. They demonstrate the importance of choosing the right strain or species for investigations of the function of a particular gene. However, it is not always possible to infer from the position of the frameshift whether the protein's activity will be affected. For instance, GlnA3, a glutamine synthetase generated from a frameshift-containing gene (Table 1), has been purified and shown to retain some activity [45]. It would be interesting to reframe these ORFs to test the impact of frameshift on protein function. On the other hand, it has been shown *in silico *that protein-coding sequences can be tolerant of frameshift translation events and thus that frameshit acquisition is an important reservoir for creating novel proteins [46]. Several of the truncated ORFs described here have also been detected in other studies, based on different analyses [17,18,40,47,48]. However, we present here a comprehensive comparative analysis of three related mycobacterial species and nine strains at the ICDS level.

We found no association between ICDS formation and mutation in the promoter region of the corresponding ORF. This suggests that promoter mutation and inactivation of gene expression are not the principal source of ICDS formation and hence of pseudogene accumulation in the *M. tuberculosis *complex. It may also suggest that ICDS formation in these species is a recent process. We favor the hypothesis that ORFs are first split into two or three parts, inactivating their function, and are then subject to secondary mutation (in both the ICDS and the untranslated region), leading to irreversible pseudogene fixation. Consistent with this hypothesis, we have observed additional mutations in the vicinity of the original frameshift in some strains.

We have shown that ICDS investigation can be used to infer the evolutionary relationships between strains and species. We provide here a list of more than 150 ICDSs that may be useful for characterizing TB strains and inferring phylogenetic relationships. The genome sequences of more than 10 TB strains will be released in the near future [26], and will, by no doubt, identify some new common and strain-specific ICDSs. Strain typing should clearly combine various markers, such as SNPs, MIRU, LSPs, RD, PE polymorphism [49] and ICDSs, in a matrix-based comparison from which the global phylogeny of TB isolates may be deduced. The polymorphism associated with these mutations is complementary to other methods [17,34,36,37,50], hence can be used to explore genetic diversity within a given species. Interestingly, in strain 210, from the W-Beijing family, eight of the 19 ICDSs common to the five *M. tuberculosis *strains tested (H37Rv, CDC1551, Haarlem, F11, C) corresponded to full-length ORFs, illustrating its earlier divergence. Some of these genes may be involved in virulence, as they concern functions such as host cell invasion (ICDS0011 of H37Rv), lipid biosynthesis (ICDS0066 and ICDS0031 of H37Rv) and intermediary metabolism (ICDS0085 of H37Rv). To test whether this trait was a particularity of the 210 strain or applied more generally to the W-Beijing phylum, we sequenced these eight ORF that were full-length in this strain in 21 other clinical isolates of the W-Beijing (Table 3). In all cases, the ORF were corresponding to a full-length ORF and not to an ICDS, demonstrating that these strains are genetically homogenous. The analysis performed using a strain of *M. africanum *showed that this species is characterized by an even fewer number of ICDSs common to *M. tuberculosis *H37Rv and CDC1551 than to the W-Beijing strains. More genome sequences of various strains and species are required for characterization of the genetic differences between the W-Beijing strains and other species of the *M. tuberculosis *complex. The *alkA *gene has been shown to contain frameshift in both *M. bovis *and some *M. tuberculosis *isolates from Central African Republic [48]. The presence of SNPs in the adjacent region of the non-sense mutation has led the authors to propose a convergent evolution. Although, it probably depends from genes to genes, we instead favor the hypothesis that the non-sense mutation was acquired by the ancestor and spread to the progeny with acquisition of subsequent mutations in the adjacent region. Epidemiologists should bear in mind that a small percentage of ICDSs may correspond to sequencing errors [4,11], generating artifactual genetic differences. Our analysis did not allow for the detection of mutations in which the frame of the coding sequence was conserved (synonymous mutation, in frame deletion), decreasing the total level of diversity observed. However, comparative ICDS analysis presents the major advantage of making it possible to associate the frameshift with a putative function and, possibly, with a particular phenotype. In conclusion, more attention should be paid to ICDS detection and comparison, particularly at the genomic scale.

## Conclusion

We report here a comparative analysis of ICDSs in six isolates of *M. tuberculosis*, two of *M. bovis *and one of *M. africanum*. We show that these ICDSs can be classified as "common to all strains" or "strain-specific". Common ICDSs result from mutations acquired before the divergence of the species, whereas strain-specific ICDSs were acquired after this divergence. Comparative analyses of these ICDSs allow the definition of the molecular signature of a particular strain, phylogenetic lineage or species. We further show that ICDS formation is not correlated with the presence of a mutated promoter, and suggest that promoter extinction is not the main cause of pseudogene formation. The correlation between ICDSs, function and phenotypes could have important evolutionary implications and provides population geneticists with a list of targets, which could undergo selective pressure and thus alters relationships between the various lineages of *M. tuberculosis *strains and their host.

## Methods

### Databases

The genome sequences of *M. tuberculosis *H37Rv and CDC1551 and *M. bovis *AF2122/97 were taken from TIGR website [51]. The genome sequences of *M. tuberculosis *strains 210 or F11, C and Haarlem have been consulted on the TIGR or Broad Institute websites [52]. The genome sequence of *M. bovis *BCG 1173P2 has been taken from National Center for Biotechnology Information (NCBI) website (accession number, AM408590). The genome sequence of *M. africanum *GM041182 was consulted on line at the Sanger centre [53].

### Detection of common ICDS

The genomic sequences of *M. tuberculosis *CDC1551, *M. tuberculosis *H37Rv, *M. bovis *AF2122/97 and *M. bovis *BCG 1173P2 have been scanned for couple of adjacent coding sequences that exhibit common homologs after translation. Such pair of coding sequences is considered as an ICDS if no paralogy relationship exists between the two coding sequences. The detailed description of ICDS detection is described in [3]. The ICDSs detected in each strain were then cross-compared by all-against-all blastn searches. For each ICDS, the best hits (E < 10^-65^) detected in the different strains were manually analysed to discriminate common and strain-specific ICDS.

### Sequencing analysis

Chromosomal DNA of *M. tuberculosis *isolates from various lineages (Table 3) was used as a template for PCR amplification of the selected locus. The primers used to amplify and sequence were designed as previously described [3], using an optimized version of CADO4MI [54]. The nucleotide and deduced amino-acid sequences were analyzed with DNA Strider [55].

### Promoter analysis

A region of 200 bp upstream the initiation codon was extracted for each of the 99 ICDSs specific to *M. tuberculosis *H37Rv, CDC1551 and *M. bovis *AF2122/97 (Additional files 2, 3 and 4). As a control group, 200 bp upstream the initiation codon was extracted for 99 genes (full-length) randomly selected from *M. tuberculosis *H37Rv. These 99 genes are full-length in *M. tuberculosis *H37Rv, CDC1551 and *M. bovis *AF2122/97. In each case (promoter to be tested and control group), the promoter regions of the 3 strains were aligned using ClustalW [56] and the sequence variation was recorded. The number of differences observed in the upstream region was statistically compared using the Chi2 test.

### Statistical analysis

The statistical significance of the distribution of the frequency of sequence polymorphism observed in the upstream ICDS regions and upstream full-length regions, was tested using a Chi square test (X^2^). The chi square test is used to determine relationship between two distributions. The calculated values were obtained: X^2^: 1,367, df: 1, P value: 0.2423, hence the difference between 2 groups are not statistically significant (α < 0.05).

## Abbreviations

ICDS, Interrupted CoDing Sequence. ORF, Open Reading Frame.

## Authors' contributions

CD helped to carry out the bioinformatic studies, analysed the TB strains by sequencing and drafted the manuscript. EP carried out the bioinformatic studies and helped to draft the manuscript. DE analysed the TB strains by sequencing. EF helped to analyze the promoter regions. OP helped to draft the manuscript. PB participated in the analysis of the W-Beijing strains and help to write the manuscript. OL participated in the design of the study, carried out the bioinformatic studies and drafted the manuscript. JMR conceived the study, participated in its design and coordination and in finalizing of the manuscript. All authors read and approved the final manuscript.

## Supplementary Material

Additional file 1 Click here for file

Additional file 2 Click here for file

Additional file 3 Click here for file

Additional file 4 Click here for file

Additional file 5 Nucleotide sequence differences of the upstream region (200 bp) of the A- strain specific ICDS B- the full-length genes (control group). Click here for file

## References

[B1] Cruveiller S Le Saux J Vallenet D Lajus A Bocs S Medigue C MICheck: a web tool for fast checking of syntactic annotations of bacterial genomes Nucleic Acids Res 2005 33 Web Server W471 479 10.1093/nar/gki498 15980515 PMC1160258

[B2] Liu Y Harrison PM Kunin V Gerstein M Comprehensive analysis of pseudogenes in prokaryotes: widespread gene decay and failure of putative horizontally transferred genes Genome Biol 2004 5 9 R64 10.1186/gb-2004-5-9-r64 15345048 PMC522871

[B3] Perrodou E Deshayes C Muller J Schaeffer C Van Dorsselaer A Ripp R Poch O Reyrat JM Lecompte O ICDS database: interrupted CoDing sequences in prokaryotic genomes Nucleic Acids Res 2006 34 Database D338 343 10.1093/nar/gkj060 16381882 PMC1347423

[B4] Deshayes C Perrodou E Gallien S Euphrasie D Schaeffer C Van-Dorsselaer A Poch O Lecompte O Reyrat JM Interrupted coding sequences in *Mycobacterium smegmatis *: authentic mutations or sequencing errors? Genome Biol 2007 8 2 R20 10.1186/gb-2007-8-2-r20 17295914 PMC1852416

[B5] Gomez-Valero L Rocha EP Latorre A Silva FJ Reconstructing the ancestor of Mycobacterium leprae: the dynamics of gene loss and genome reduction Genome Res 2007 17 8 1178 1185 10.1101/gr.6360207 17623808 PMC1933519

[B6] Cole ST Eiglmeier K Parkhill J James KD Thomson NR Wheeler PR Honore N Garnier T Churcher C Harris D Massive gene decay in the leprosy bacillus Nature 2001 409 6823 1007 1011 10.1038/35059006 11234002

[B7] Stinear TP Mve-Obiang A Small PL Frigui W Pryor MJ Brosch R Jenkin GA Johnson PD Davies JK Lee RE Giant plasmid-encoded polyketide synthases produce the macrolide toxin of Mycobacterium ulcerans Proc Natl Acad Sci USA 2004 101 5 1345 1349 10.1073/pnas.0305877101 14736915 PMC337055

[B8] Darby AC Cho NH Fuxelius HH Westberg J Andersson SG Intracellular pathogens go extreme: genome evolution in the Rickettsiales Trends Genet 2007 23 10 511 520 10.1016/j.tig.2007.08.002 17822801

[B9] Guan X Uberbacher EC Alignments of DNA and protein sequences containing frameshift errors Comput Appl Biosci 1996 12 1 31 40 8670617 10.1093/bioinformatics/12.1.31

[B10] Hayashi K Morooka N Yamamoto Y Fujita K Isono K Choi S Ohtsubo E Baba T Wanner BL Mori H Highly accurate genome sequences of Escherichia coli K-12 strains MG1655 and W3110 Mol Syst Biol 2006 2 2006 0007. 10.1038/msb4100049 16738553 PMC1681481

[B11] Medigue C Rose M Viari A Danchin A Detecting and analyzing DNA sequencing errors: toward a higher quality of the *Bacillus subtilis *genome sequence Genome Res 1999 9 11 1116 1127 10.1101/gr.9.11.1116 10568751 PMC310837

[B12] Xu Y Mural RJ Uberbacher EC Correcting sequencing errors in DNA coding regions using a dynamic programming approach Comput Appl Biosci 1995 11 2 117 124 7620982 10.1093/bioinformatics/11.2.117

[B13] Friedman CR Quinn GC Kreiswirth BN Perlman DC Salomon N Schluger N Lutfey M Berger J Poltoratskaia N Riley LW Widespread dissemination of a drug-susceptible strain of *Mycobacterium tuberculosis* J Infect Dis 1997 176 2 478 484 9237715 10.1086/514067

[B14] Mathema B Kurepina NE Bifani PJ Kreiswirth BN Molecular epidemiology of tuberculosis: current insights Clin Microbiol Rev 2006 19 4 658 685 10.1128/CMR.00061-05 17041139 PMC1592690

[B15] Gutacker MM Mathema B Soini H Shashkina E Kreiswirth BN Graviss EA Musser JM Single-nucleotide polymorphism-based population genetic analysis of *Mycobacterium tuberculosis *strains from 4 geographic sites J Infect Dis 2006 193 1 121 128 10.1086/498574 16323140

[B16] Sreevatsan S Pan X Stockbauer KE Connell ND Kreiswirth BN Whittam TS Musser JM Restricted structural gene polymorphism in the Mycobacterium tuberculosis complex indicates evolutionarily recent global dissemination Proc Natl Acad Sci USA 1997 94 18 9869 9874 10.1073/pnas.94.18.9869 9275218 PMC23284

[B17] Filliol I Motiwala AS Cavatore M Qi W Hazbon MH Bobadilla del Valle M Fyfe J Garcia-Garcia L Rastogi N Sola C Global phylogeny of Mycobacterium tuberculosis based on single nucleotide polymorphism (SNP) analysis: insights into tuberculosis evolution, phylogenetic accuracy of other DNA fingerprinting systems, and recommendations for a minimal standard SNP set J Bacteriol 2006 188 2 759 772 10.1128/JB.188.2.759-772.2006 16385065 PMC1347298

[B18] Fleischmann RD Alland D Eisen JA Carpenter L White O Peterson J DeBoy R Dodson R Gwinn M Haft D Whole-genome comparison of Mycobacterium tuberculosis clinical and laboratory strains J Bacteriol 2002 184 19 5479 5490 10.1128/JB.184.19.5479-5490.2002 12218036 PMC135346

[B19] Manca C Tsenova L Barry CE 3rd Bergtold A Freeman S Haslett PA Musser JM Freedman VH Kaplan G *Mycobacterium tuberculosis *CDC1551 induces a more vigorous host response in vivo and in vitro, but is not more virulent than other clinical isolates J Immunol 1999 162 11 6740 6746 10352293

[B20] Manca C Tsenova L Bergtold A Freeman S Tovey M Musser JM Barry CE 3rd Freedman VH Kaplan G Virulence of a *Mycobacterium tuberculosis *clinical isolate in mice is determined by failure to induce Th1 type immunity and is associated with induction of IFN-alpha/beta Proc Natl Acad Sci USA 2001 98 10 5752 5757 10.1073/pnas.091096998 11320211 PMC33285

[B21] Reed MB Domenech P Manca C Su H Barczak AK Kreiswirth BN Kaplan G Barry CE 3rd A glycolipid of hypervirulent tuberculosis strains that inhibits the innate immune response Nature 2004 431 7004 84 87 10.1038/nature02837 15343336

[B22] Camus JC Pryor MJ Medigue C Cole ST Re-annotation of the genome sequence of *Mycobacterium tuberculosis *H37Rv Microbiology 2002 148 Pt 10 2967 2973 12368430 10.1099/00221287-148-10-2967

[B23] Cole ST Brosch R Parkhill J Garnier T Churcher C Harris D Gordon SV Eiglmeier K Gas S Barry CE 3rd Deciphering the biology of *Mycobacterium tuberculosis *from the complete genome sequence Nature 1998 393 6685 537 544 10.1038/31159 9634230

[B24] Garnier T Eiglmeier K Camus JC Medina N Mansoor H Pryor M Duthoy S Grondin S Lacroix C Monsempe C The complete genome sequence of *Mycobacterium bovis* Proc Natl Acad Sci USA 2003 100 13 7877 7882 10.1073/pnas.1130426100 12788972 PMC164681

[B25] Brosch R Gordon SV Marmiesse M Brodin P Buchrieser C Eiglmeier K Garnier T Gutierrez C Hewinson G Kremer K A new evolutionary scenario for the Mycobacterium tuberculosis complex Proc Natl Acad Sci USA 2002 99 6 3684 3689 10.1073/pnas.052548299 11891304 PMC122584

[B26] Bernal A Ear U Kyrpides N Genomes OnLine Database (GOLD): a monitor of genome projects world-wide Nucleic Acids Res 2001 29 1 126 127 10.1093/nar/29.1.126 11125068 PMC29859

[B27] Brosch R Gordon SV Garnier T Eiglmeier K Frigui W Valenti P Dos Santos S Duthoy S Lacroix C Garcia-Pelayo C Genome plasticity of BCG and impact on vaccine efficacy Proc Natl Acad Sci USA 2007 104 13 5596 5601 10.1073/pnas.0700869104 17372194 PMC1838518

[B28] Oettinger T Jorgensen M Ladefoged A Haslov K Andersen P Development of the *Mycobacterium bovis *BCG vaccine: review of the historical and biochemical evidence for a genealogical tree Tuber Lung Dis 1999 79 4 243 250 10.1054/tuld.1999.0206 10692993

[B29] Brosch R Gordon SV Buchrieser C Pym AS Garnier T Cole ST Comparative genomics uncovers large tandem chromosomal duplications in *Mycobacterium bovis *BCG Pasteur Yeast 2000 17 2 111 123 10.1002/1097-0061(20000630)17:2<111::AID-YEA17>3.0.CO;2-G 10900457 PMC2448323

[B30] Pym AS Brodin P Brosch R Huerre M Cole ST Loss of RD1 contributed to the attenuation of the live tuberculosis vaccines *Mycobacterium bovis *BCG and *Mycobacterium microti* Mol Microbiol 2002 46 3 709 717 10.1046/j.1365-2958.2002.03237.x 12410828

[B31] Lopez B Aguilar D Orozco H Burger M Espitia C Ritacco V Barrera L Kremer K Hernandez-Pando R Huygen K A marked difference in pathogenesis and immune response induced by different *Mycobacterium tuberculosis *genotypes Clin Exp Immunol 2003 133 1 30 37 10.1046/j.1365-2249.2003.02171.x 12823275 PMC1808750

[B32] Mira A Pushker R The silencing of pseudogenes Mol Biol Evol 2005 22 11 2135 2138 10.1093/molbev/msi209 16014873

[B33] Bocs S Danchin A Medigue C Re-annotation of genome microbial coding-sequences: finding new genes and inaccurately annotated genes BMC Bioinformatics 2002 3 5 10.1186/1471-2105-3-5 11879526 PMC77393

[B34] Goguet de la Salmoniere YO Li HM Torrea G Bunschoten A van Embden J Gicquel B Evaluation of spoligotyping in a study of the transmission of *Mycobacterium tuberculosis* J Clin Microbiol 1997 35 9 2210 2214 9276389 10.1128/jcm.35.9.2210-2214.1997 PMC229941

[B35] Kamerbeek J Schouls L Kolk A van Agterveld M van Soolingen D Kuijper S Bunschoten A Molhuizen H Shaw R Goyal M Simultaneous detection and strain differentiation of *Mycobacterium tuberculosis *for diagnosis and epidemiology J Clin Microbiol 1997 35 4 907 914 9157152 10.1128/jcm.35.4.907-914.1997 PMC229700

[B36] Mazars E Lesjean S Banuls AL Gilbert M Vincent V Gicquel B Tibayrenc M Locht C Supply P High-resolution minisatellite-based typing as a portable approach to global analysis of Mycobacterium tuberculosis molecular epidemiology Proc Natl Acad Sci USA 2001 98 4 1901 1906 10.1073/pnas.98.4.1901 11172048 PMC29354

[B37] Kaser M Rondini S Naegeli M Stinear T Portaels F Certa U Pluschke G Evolution of two distinct phylogenetic lineages of the emerging human pathogen Mycobacterium ulcerans BMC Evol Biol 2007 7 1 177 10.1186/1471-2148-7-177 17900363 PMC2098775

[B38] Constant P Perez E Malaga W Laneelle MA Saurel O Daffe M Guilhot C Role of the pks15/1 gene in the biosynthesis of phenolglycolipids in the *Mycobacterium tuberculosis *complex. Evidence that all strains synthesize glycosylated p-hydroxybenzoic methyl esters and that strains devoid of phenolglycolipids harbor a frameshift mutation in the pks15/1 gene J Biol Chem 2002 277 41 38148 38158 10.1074/jbc.M206538200 12138124

[B39] Gagneux S DeRiemer K Van T Kato-Maeda M de Jong BC Narayanan S Nicol M Niemann S Kremer K Gutierrez MC Variable host-pathogen compatibility in *Mycobacterium tuberculosis* Proc Natl Acad Sci USA 2006 103 8 2869 2873 10.1073/pnas.0511240103 16477032 PMC1413851

[B40] Tsolaki AG Gagneux S Pym AS Goguet de la Salmoniere YO Kreiswirth BN Van Soolingen D Small PM Genomic deletions classify the Beijing/W strains as a distinct genetic lineage of *Mycobacterium tuberculosis* J Clin Microbiol 2005 43 7 3185 3191 10.1128/JCM.43.7.3185-3191.2005 16000433 PMC1169157

[B41] Reed MB Gagneux S Deriemer K Small PM Barry CE 3rd The W-Beijing lineage of *Mycobacterium tuberculosis *overproduces triglycerides and has the DosR dormancy regulon constitutively upregulated J Bacteriol 2007 189 7 2583 2589 10.1128/JB.01670-06 17237171 PMC1855800

[B42] Perez E Constant P Lemassu A Laval F Daffe M Guilhot C Characterization of three glycosyltransferases involved in the biosynthesis of the phenolic glycolipid antigens from the *Mycobacterium tuberculosis *complex J Biol Chem 2004 279 41 42574 42583 10.1074/jbc.M406246200 15292272

[B43] Boechat N Lagier-Roger B Petit S Bordat Y Rauzier J Hance AJ Gicquel B Reyrat JM Disruption of the gene homologous to mammalian Nramp1 in *Mycobacterium tuberculosis *does not affect virulence in mice Infect Immun 2002 70 8 4124 4131 10.1128/IAI.70.8.4124-4131.2002 12117920 PMC128187

[B44] Domenech P Reed MB Barry CE 3rd Contribution of the *Mycobacterium tuberculosis *MmpL protein family to virulence and drug resistance Infect Immun 2005 73 6 3492 3501 10.1128/IAI.73.6.3492-3501.2005 15908378 PMC1111821

[B45] Harth G Maslesa-Galic S Tullius MV Horwitz MA All four *Mycobacterium tuberculosis *glnA genes encode glutamine synthetase activities but only GlnA1 is abundantly expressed and essential for bacterial homeostasis Mol Microbiol 2005 58 4 1157 1172 10.1111/j.1365-2958.2005.04899.x 16262797

[B46] Okamura K Feuk L Marques-Bonet T Navarro A Scherer SW Frequent appearance of novel protein-coding sequences by frameshift translation Genomics 2006 88 6 690 697 10.1016/j.ygeno.2006.06.009 16890400

[B47] Marri PR Bannantine JP Golding GB Comparative genomics of metabolic pathways in Mycobacterium species: gene duplication, gene decay and lateral gene transfer FEMS Microbiol Rev 2006 30 6 906 925 10.1111/j.1574-6976.2006.00041.x 17064286

[B48] Nouvel LX Dos Vultos T Kassa-Kelembho E Rauzier J Gicquel B A non-sense mutation in the putative anti-mutator gene ada/alkA of Mycobacterium tuberculosis and M. bovis isolates suggests convergent evolution BMC Microbiol 2007 7 39 10.1186/1471-2180-7-39 17506895 PMC1891112

[B49] Karboul A Gey van Pittius NC Namouchi A Vincent V Sola C Rastogi N Suffys P Fabre M Cataldi A Huard RC Insights into the evolutionary history of tubercle bacilli as disclosed by genetic rearrangements within a PE_PGRS duplicated gene pair BMC Evol Biol 2006 6 107 10.1186/1471-2148-6-107 17163995 PMC1762029

[B50] Read TD Salzberg SL Pop M Shumway M Umayam L Jiang L Holtzapple E Busch JD Smith KL Schupp JM Comparative genome sequencing for discovery of novel polymorphisms in Bacillus anthracis Science 2002 296 5575 2028 2033 10.1126/science.1071837 12004073

[B51] J. Craig Venter Institute http://www.tigr.org/

[B52] The BROAD Institute http://www.broad.mit.edu/tools/data/seq.html

[B53] Welcome trust Sanger Institute Mycobacterium africanum http://www.sanger.ac.uk/sequencing/Mycobacterium/africanum/

[B54] Computer assisted Design of Oligonucleotide for Microarray http://bips.u-strasbg.fr/CADO4MI/

[B55] Marck C 'DNA Strider': a 'C' program for the fast analysis of DNA and protein sequences on the Apple Macintosh family of computers Nucleic Acids Res 1988 16 5 1829 1836 10.1093/nar/16.5.1829 2832831 PMC338177

[B56] Thompson JD Higgins DG Gibson TJ CLUSTAL W: improving the sensitivity of progressive multiple sequence alignment through sequence weighting, position-specific gap penalties and weight matrix choice Nucleic Acids Res 1994 22 22 4673 4680 10.1093/nar/22.22.4673 7984417 PMC308517

