# Supplementary material for: Detecting the molecular scars of evolution in the Mycobacterium tuberculosis complex by analyzing interrupted coding sequences
Source: BMC Evol Biol. 2008 Mar 6;8:78. doi: 10.1186/1471-2148-8-78 (PMC2277376; doi:10.1186/1471-2148-8-78)
Supplement: Additional file 1 [file 1471-2148-8-78-S1.doc]

**Additional Table 1.**

| ***M. tuberculosis***  **H37Rv** | ***M. tuberculosis* CDC1551** | ***M. bovis* AF2122/97** | **Putative function** | ***M. tuberculosis***  **Haarlem** | ***M. tuberculosis***  **C** | ***M. tuberculosis***  **F11** |
| --- | --- | --- | --- | --- | --- | --- |
| 0002 (Rv0151c) | 0004 | 0006 | PE family protein |  | A-C at 177177 |  |
| 0003 (Rv0152c) | 0005 | 0007 | PE family protein |  | C-T at 179994 | C-T at 180025  **G-E in Rv0152c** |
| 0009 (Rv0393) | 0012 | 0011 | Conserved hypothetical |  |  | T-C at 472705 |
| 0012 (Rv0601c) | 0017 | 0018 | Two-component sensor kinase | G-A at 698968 | G-A at 698968 | G-A at 698968 |
| 0026 (Rv1104) | 0034 | 0087 | Esterase | C-A at 1232037 |  |  |
| 0027 (Rv1104) | 0035 | 0088 | Esterase | C-A at 1232037 |  |  |
| 0028 | 0036 | 0033 | Esterase |  |  | T-C at 1232496 |
| 0030 (Rv1136) | 0039 | 0089 | Enoyl-CoA |  |  | CGC added at 1264989 |
| 0036 (Rv1413) | 0046 | 0041 | Conserved hypothetical | A-G at 1589383 | A-G at 1589383 |  |
| 0065 (Rv2943A-Rv2944) | 0083 | 0066 | Transposase | C-A at 3289923  **P-H in Rv2944** | C-A at 3289923  **P-H in Rv2944** |  |
| 0075 (Rv3349c) | 0100 | 0102 | Transposase |  | C-T at 3755066 |  |
| 0080 (Rv3421c) | 0105 | 0104 | Conserved hypothetical | A-G at 3838871 | A-G at 3838871 | A-G at 3838871 |
| 0091 (Rv3880c-Rv3881c) | 0118 | 0137 | Conserved hypothetical |  | C-T at 4360512  **R-Q in Rv3880c**  T-G at 4360536  **D-A in Rv3880c** |  |
| 0103 (Rv0449c) | 0129 | 0113 | Conserved hypothetical |  | G deleted at 537344  **Full-length**  G deleted at 537395 |  |
| 0105 (Rv0859-Rv0860) | 0131 | 0115 | Acyl-CoA thiolase FadA and dehydrogenase FadB | A-G at 955524  **S-G in Rv0859**  T-C at 957117 | A-G at 955524  **S-G in Rv0859**  C-T at 956108  **P-L in Rv0859**  C deleted at 956155  **Full-length**  C deleted at 956220  T-C at 957117 | A-G at 955524  **S-G in Rv0859** |
| 0106 (Rv0880-Rv0881) | 0132 | 0116 | Transcriptional regulator | G-C at 979704  **G-R in Rv0881** | G-C at 979704  **G-R in Rv0881** | G-C at 979704  **G-R in Rv0881** |
| 0108 (Rv1041c-Rv1042c) | 0134 | 0118 | Transposase | G-A at 1164336 | G-A at 1164336  G-A at 1164361 |  |
| 0113 (Rv2309c) | 0139 | 0123 | Integrase |  | G-T at 2582348 |  |
| 0117 (Rv3774-Rv3775) | 0143 | 0127 | Enoyl-CoA hydratase EchA21 and lipase LipE |  |  | G-A at 422017  **D-N in Rv3775** |
| 0001 (RV0095c) | 0003 | | Conserved hypothetical | C-T at 104712 | C-T at 104712  TGGG-CCGA at 104941  **QA-GV in Rv0095c** | Not found |
| 0013 (Rv0618-Rv0619) | 0018 | | Galactose-1-phosphate uridylyltransferase |  | G-A at 712160  **E-K in Rv0618** |  |
| 0039 (Rv1553-Rv1554) | 0052 | | Fumarate reductase | A-G at 1760292  **M-V in Rv1554** | G-A at 1760043  **Shorter Rv1553**  G-C at 1760058  C-T at 1760088  G-C at 1760173  C added at 1760175  A-G at 1760293  **V-M in Rv1554**  G-C at 1760320  **R-G in Rv1554** | A-G at 1760292  **M-V in Rv1554** |
| 0045 (Rv1792) | 0058 | | ESAT-6-like protein EsxM |  |  | G added at 2030342  A-G at 2030345  A-G at 2030355 |
| 0067 (Rv2974c-Rv2975c) | 0085 | | Conserved hypothetical |  | C-T at 3331171  T added at 3331173  **Variation in Rv2974c C-ter**  C-T at 3331186  **G-D in Rv2974c** |  |
| 0072 (Rv3233c-Rv3234c | 0092 | | Conserved hypothetical |  |  | C-T at 3610441  **R-H in Rv3234c** |
| 0074 (Rv3349c) | 0099 | | Transposase |  | C-T at 3755066 |  |
| 0086 (Rv3738c-Rv3739c) | 0113 | | PPE family protein | A-G at 4190332 |  |  |

List of the ICDSs containing additional mutations in the *M. tuberculosis* strains Haarlem, C and F11 with respect to H37Rv genome sequence. The amino acid mutations are indicated in bold.
