# Supplementary material for: Detecting the molecular scars of evolution in the Mycobacterium tuberculosis complex by analyzing interrupted coding sequences
Source: BMC Evol Biol. 2008 Mar 6;8:78. doi: 10.1186/1471-2148-8-78 (PMC2277376; doi:10.1186/1471-2148-8-78)
Supplement: Additional file 2 [file 1471-2148-8-78-S2.doc]

**Additional Table 2.**

| ***M. bovis***  **AF2122/97** | ***M. tuberculosis***  **H37Rv** | ***M. tuberculosis***  **CDC1551** | **Putative function** | **Functional classification** |
| --- | --- | --- | --- | --- |
| 0001^$^ (Mb0074 *280 aa* - Mb0075 *70 aa*) | Rv0073 *330 aa* | MT0079 *330 aa* | Glutamine-transport ABC transporter GlnQ | Cell wall, process |
| 0002^$^ (Mb0124c *597 aa* - Mb0125c *117 aa*) | Rv0120c *714 aa* | MT0128 *714 aa* | Elongation factor G | Information pathway |
| 0003^$^ (Mb0139.1 *128 aa* - Mb0139.2 *36 aa*) | Rv0134 *300 aa* | MT0142 *300 aa* | Epoxide hydrolase EphF | Virulence, detox, adapt |
| 0004 (Mb0139.2 *36 aa* - Mb0139.3 *80 aa*) | Rv0134 *300 aa* | MT0142 *300 aa* | Epoxide hydrolase EphF | Virulence, detox, adapt |
| 0005^$^ (Mb0139.3 *80 aa* - Mb0139.4 *55 aa*) | Rv0134 *300 aa* | MT0142 *300 aa* | Epoxide hydrolase EphF | Virulence, detox, adapt |
| 0008 (Mb0228c *385 aa*) | Rv0223c *487 aa* | MT0233 *487 aa* | Aldehyde dehydrogenase | Intermediary metabolism |
| 0012 (Mb0412 *460 aa* - Mb0413 *946 aa*) | Rv0405 *1402 aa* | MT0418 *1402 aa* | Polyketide synthase Pks6 | Lipid metabolism |
| 0014 (Mb0563c *45 aa* - Mb0564c *102 aa*) | Rv0549c *137 aa* | MT0574 *137 aa* | Conserved hypothetical | Unknown |
| 0015 (Mb0585 *710 aa*) | Rv0570 *692 aa* | MT0596 *735 aa* | Ribonucleoside-diphosphate reductase NdrZ | Information pathway |
| 0016 (Mb0590c *378 aa*) | Rv0575c *388 aa* | MT0604 *388 aa* | Oxidoreductase | Intermediary metabolism |
| 0017 (Mb0609 *188 aa*) | Rv0593 *402 aa* | MT0623 *402 aa* | MCE-family protein | Virulence, detox, adapt |
| 0019 (Mb0646c *234 aa* - Mb0647c *897 aa*) | Rv0630c *1094 aa* | MT0658 *1094 aa* | Exonuclease V RecB | Information pathway |
| 0022^$^ (Mb0731 *148 aa* - Mb0732 *636 aa*) | Rv0711 *787 aa* | MT0738 *757 aa* | Arylsulfatase AtsA | Intermediary metabolism |
| 0024 (Mb0818c *203 aa* - Mb0819c *299 aa*) | Rv0794c *499 aa* | MT0817 *643 aa* | Oxidoreductase | Intermediary metabolism |
| 0026 (Mb0954c *369 aa* - Mb0955c *291 aa*) | Rv0931c *664 aa* | MT0958 *664 aa* | Serine/threonine protein kinase PknD | Regulatory |
| 0027 (Mb0957 *71 aa* - Mb0958 *213 aa*) | Rv0933 *276 aa* | MT0960 *276 aa* | phosphate-transport ABC transporter PstB | Cell wall, process |
| 0028^$^ (Mb1013 *423 aa* - Mb1014 *431 aa*) | Rv0987 *855 aa* | MT1015 *855 aa* | Adhesion component transport ABC transporter | Cell wall, process |
| 0029 (Mb1044c *252 aa*) | Rv1016c *226 aa* | MT1044 *226 aa* | Conserved lipoprotein LpqT | Cell wall, process |
| 0037 (Mb1290 *274 aa* - Mb1291 *102 aa*) | Rv1260 *372 aa* | MT1298 *383 aa* | Oxidoreductase | Intermediary metabolism |
| 0038^$^ (Mb1345c *243 aa* - Mb1346c *205 aa*) | Rv1313c *444 aa* | MT1353 *444 aa* | Transposase | IS/phage |
| 0039^$^ (Mb1350c *416 aa* - Mb1351c *78 aa*) | Rv1317c *496 aa* | MT1358 *496 aa* | Adapative response regulatory protein AlkA | Information pathway |
| 0040^$^ (Mb1407 *265 aa* - Mb1408 *172 aa*) | Rv1373 *326 aa* | MT1418 *320 aa* | Glycolipid sulfotransferase | Intermediary metabolism |
| 0042^$^ (Mb1540 *116 aa* - Mb1541 *189 aa*) | Rv1502 *299 aa* | MT1551 *299 aa* | Hypothetical | Unknown |
| 0044 (Mb1746 *207 aa*) | Rv1718 *272 aa* | NP | Conserved hypothetical | Unknown |
| 0045 (Mb1785c *394 aa*) | NP | MT1800 *381 aa* | Glycosyltransferase | Intermediary metabolism |
| 0047 (Mb1791 *509 aa*) | Rv1760 *502 aa* | MT1809 *531 aa* | Conserved hypothetical | Unknown |
| 0048 (Mb1831c *540 aa* - Mb1832c *87 aa*) | Rv1803c *639 aa* | MT1853 *650 aa* | PE-PGRS family protein | PE/PPE |
| 0049 (Mb1838 *187 aa* - Mb1839 *282 aa*) | Rv1809 *468 aa* | MT1857 *694 aa* | PPE family protein | PE/PPE |
| 0050 (Mb1875c *685 aa*) | Rv1844c *485 aa* | MT1892 *483 aa* | 6-phosphogluconate dehydrogenase Gnd1 | Intermediary metabolism |
| 0051 (Mb1908 *511 aa* - Mb1909 *404 aa*) | Rv1877 *687 aa* | MT1926 *687 aa* | Conserved hypothetical | Unknown |
| 0057^$^ (Mb2055c *79 aa* - Mb2056c *606 aa*) | Rv2030c *681 aa* | MT2089 *681 aa* | Conserved hypothetical | Unknown |
| 0059 (Mb2307c *87 aa* - Mb2308c *133 aa*) | Rv2286c *230 aa* | MT2344 *230 aa* | Conserved hypothetical | Unknown |
| 0061 (Mb2367 *826 aa* - Mb2368 *131 aa*) | Rv2339 *962 aa* | MT2402 *962 aa* | Transmembrane transport protein MmpL9 | Cell wall, process |
| 0062 (Mb2595 *533 aa* - Mb2596 *597 aa*) | Rv2566 *1140 aa* | MT2642 *1156 aa* | Transglutaminase | Intermediary metabolism |
| 0063 (Mb2762c *82 aa* - Mb2763c *195 aa*) | Rv2742c *277 aa* | MT2813 *286 aa* | Conserved hypothetical | Unknown |
| 0064^$^ (Mb2859c *180 aa* - Mb2860c *123 aa*) | Rv2835c *303 aa* | MT2901 *303 aa* | Sn-glycerol-3-phosphate-binding lipoprotein UgpA | Cell wall, process |
| 0067 (Mb2982c *366 aa*) | Rv2958c *428 aa* | MT3034 *428 aa* | Glycosyltransferase | Intermediary metabolism |
| 0070 (Mb3201c *105 aa* - Mb3202c *208 aa*) | Rv3176c *318 aa* | MT3265 *339 aa* | Epoxide hydrolase MesT | Virulence, detox, adapt |
| 0073 (Mb3507 *433 aa* - Mb3508 *348 aa*) | Rv3479 *1021 aa* | MT3583 *1075 aa* | Conserved hypothetical | Unknown |
| 0074^$^ (Mb3509c *274 aa* - Mb3510c *217 aa*) | Rv3480c *497 aa* | MT3584 *505 aa* | Conserved hypothetical | Unknown |
| 0075 (Mb3547c *193 aa* - Mb3548c *205 aa*) | Rv3518c *398 aa* | MT3619 *372 aa* | Cytochrome P450 monooxygenase1 Cyp142 | Intermediary metabolism |
| 0077 (Mb3712c *81 aa*) | Rv3687c *122 aa* | MT3789 *122 aa* | Anti-anti-sigma factor RsfB | Information pathway |
| 0080 (Mb3827 *205 aa* - Mb3828 *243 aa*) | Rv3798 *444 aa* | MT3905 *444 aa* | Transposase | IS/phage |
| 0081 (Mb3923c *561 aa* - Mb3924c *833 aa*) | Rv3894c *1396 aa* | MT4010 *1396 aa* | FtsK/SpoIIIE family protein | Cell wall, process |
| 0082 (Mb3926c *288 aa*) | Rv3896c *302 aa* | MT4012 *302 aa* | Conserved hypothetical | Unknown |
| 0091 (Mb1200c *181 aa* - Mb1201c *180 aa*) | Rv1168c *346 aa* | MT1205 *346 aa* | PPE family protein | PE/PPE |
| 0095^$^ (Mb2314 *256 aa*) | Rv2291 *284 aa* | MT2348 *268 aa* | Thiosulfate sulfurtransferase SseB | Intermediary metabolism |
| 0105^$^ (Mb3507 *433 aa*) | Rv3479 *1021 aa* | MT3583 *1075 aa* | Hypothetical | Unknown |
| 0106^$^ (Mb3507 *433 aa* - Mb3508 *348 aa*) | Rv3479 *1021 aa* | MT3583 *1075 aa* | Hypothetical | Unknown |
| 0110 (Mb3935c *57 aa*) | Rv3905c *103 aa* | MT4024 *103 aa* | ESAT-6 like protein EsxF | Cell wall, process |
| 0131 (Mb3436c *350 aa*) | Rv3402c *412 aa* | MT3510 *412 aa* | Aminotransferase | Information pathway |

List of the ICDSs specific to *M. bovis* AF2122/97 (corresponding to full-length ORF in *M. tuberculosis* H37Rv and in *M. tuberculosis* H37Rv). ICDS number, the affected ORF, the size of the predicted protein and its putative function are indicated. “$” indicates ICDSs that correspond to full-length genes in *M. bovis* BCG 1173 P2. The size (in amino acid) of the corresponding predicted protein in the genome of *M. tuberculosis* H37Rv and CDC1551 is indicated. “NP”, Not Predicted.
