# Supplementary material for: Detecting the molecular scars of evolution in the Mycobacterium tuberculosis complex by analyzing interrupted coding sequences
Source: BMC Evol Biol. 2008 Mar 6;8:78. doi: 10.1186/1471-2148-8-78 (PMC2277376; doi:10.1186/1471-2148-8-78)
Supplement: Additional file 3 [file 1471-2148-8-78-S3.doc]

**Additional Table 3.**

| ***M. tuberculosis* H37Rv** | ***M. tuberculosis* CDC15551** | ***M. bovis***  **AF2122/97** | **Putative function** | **Functional classification** |
| --- | --- | --- | --- | --- |
| 0006 (Rv0354c *141 aa)* | MT0369 *185 aa* | Mb0362c *3507 aa* | PPE family protein | PE/PPE |
| 0008 (Rv0387c *244 aa* - Rv0388c *180 aa*) | MT0400 *443 aa* | Mb0394c *443 aa* | PPE family protein | PE/PPE |
| 0018 (Rv0781 *236 aa* - Rv0782 *552 aa*) | MT0805 *718 aa* | Mb0804 *719 aa* | Protease II | Intermediary metabolism |
| 0034 (Rv1180 *488 aa* - Rv1181 *1582 aa*) | MT1218 *2101 aa* | Mb1213 *2085 aa* | Polyketide synthase Pks3/4 | Lipid metabolism |
| 0044 (Rv1783 *435 aa* - Rv1784 *932 aa*) | MT1833 *1391 aa* | Mb1218 *1391 aa* | FtsK/SpoIIIE family protein | Cell wall, process |
| 0056 (Rv2250A *139 aa* - Rv2251 *475 aa*) | MT2311 *529 aa* | Mb2275 *529 aa* | Flavoprotein | Intermediary metabolism |
| 0057 (Rv2261c *140 aa* - Rv2262c *360 aa*) | MT2322 *502 aa* | Mb2285c *502 aa* | Conserved hypothetical | Unknown |
| 0064 (Rv2879c *189 aa* - Rv2880c *275 aa*) | MT2947 *364 aa* | Mb2904c *364 aa* | Conserved hypothetical | Unknown |
| 0081 (Rv3425 *176 aa* -Rv3426 *232 aa*) | MT3533 *178 aa* – NP | Mb3459 *178 aa* - NP | PPE family protein | PE/PPE |
| 0082 (Rv3453 *110 aa* - Rv3454 *422 aa*) | MT3561 *562 aa* | Mb3483 *561 aa* | Conserved hypothetical | Unknown |
| 0092 (Rv3897c *210 aa*) | MT4013 *214 aa* | Mb3927c *329 aa* | Conserved hypothetical | Unknown |
| 0096 (Rv3911 *222 aa*) | MT4030 *196 aa* | Mb3941 *196 aa* | Alternative sigma factor SigM | Information pathway |

List of the ICDSs specific to *M. tuberculosis* H37Rv (corresponding to full-length ORF in *M. tuberculosis* CDC1551 and in *M. bovis* AF2122/97). The ICDS number, the affected ORF, the size of the predicted protein and its putative function are indicated. The size (in amino acid) of the corresponding predicted protein in the genome of *M. tuberculosis* CDC1551 and *M. bovis* AF2122/97 is indicated. “NP”, Not Predicted.
