# Supplementary material for: Detecting the molecular scars of evolution in the Mycobacterium tuberculosis complex by analyzing interrupted coding sequences
Source: BMC Evol Biol. 2008 Mar 6;8:78. doi: 10.1186/1471-2148-8-78 (PMC2277376; doi:10.1186/1471-2148-8-78)
Supplement: Additional file 4 [file 1471-2148-8-78-S4.doc]

**Additional Table 4.**

| ***M. tuberculosis***  **CDC1551** | ***M. tuberculosis***  **H37Rv** | ***M. bovis***  **AF2122/97** | **Putative function** | **Functional classification** |
| --- | --- | --- | --- | --- |
| 0001 (MT0028 *90 aa*) | Rv0025 *120 aa* | Mb0026 *120 aa* | Hypothetical | Unknown |
| 0002 (MT0032 *192 aa*) | NP | NP | Hypothetical | Unknown |
| 0006 (MT0170 *267 aa*) | Rv0161 *449 aa* | Mb0166 *449 aa* | Oxidoreductase | Intermediary metabolism |
| 0008 (MT0279 *975 aa*) | Rv0266c *1209 aa* | Mb0272c *1209 aa* | 5-oxo-L-prolinase | Intermediary metabolism |
| 0009 (MT0328 *61 aa*) | Rv0314c *220 aa* | Mb0322c *220* *aa* | Hypothetical | Unknown |
| 0013 (MT0511 *480 aa* – MT0512 *225 aa*) | Rv0492c *629 aa* | Mb0502c *629 aa* | Oxidoreductase | Intermediary metabolism |
| 0015 (MT0566 *493 aa*) | Rv0541c *449 aa* | Mb0555c *449 aa* | Conserved hypothetical | Unknown |
| 0023 (MT0765 *82 aa* – MT0766 *120 aa*) | Rv0740 *175 aa* | Mb0761 *175 aa* | Hypothetical | Unknown |
| 0024 (MT0871 *326 aa*) | Rv0848 *372 aa* | Mb0871 *372 aa* | Cysteine synthase | Intermediary metabolism |
| 0025 (MT0872 *282 aa*) | Rv0849 *419 aa* | Mb0872 *419 aa* | Conserved hypothetical | Unknown |
| 0029 (MT1004 *709 aa*) | Rv0977 *923 aa* | Mb01002 *923 aa* | PE_PGRS family protein | PE/PPE |
| 0033 (MT1099 *603 aa*) | Rv1069c *587 aa* | Mb1098c *587 aa* | Hypothetical | Unknown |
| 0038 (MT1157 *193 aa*) | Rv1125 *414 aa* | Mb1156 *414 aa* | Hypothetical | Unknown |
| 0043 (MT1216 *256 aa* – MT1217 *701 aa*) | Rv1179c *939 aa* | Mb1212c *939 aa* | Hypothetical | Unknown |
| 0045 (MT1408 *197 aa* – MT1409 *74 aa*) | Rv1363c *261 aa* | Mb1398c *261 aa* | Hypothetical | Unknown |
| 0047 (MT1469 *471 aa*) | Rv1426c *420 aa* | Mb1461c *420 aa* | Carboxylesterase | Intermediary metabolism |
| 0049 (MT1558 *172 aa* – MT1560 *256 aa*) | Rv1510 *432 aa* | NP | Hypothetical | Unknown |
| 0050 (MT1578 *1144 aa*) | Rv1527c *2108 aa* | Mb1554c *2108 aa* | Polyketide synthase Pks5 | Lipid metabolism |
| 0057* (MT1806 *820 aa* - MT1807 *94 aa*) | Rv1759c *914 aa* | Mb1789c *820 aa* – Mb1790c *94 aa* | PE_PGRS family protein | PE/PPE |
| 0059 (MT1865 *466 aa*) | Rv1817 *487 aa* | Mb1848 *487 aa* | Hypothetical | Unknown |
| 0060 (MT1883 *613 aa*) | Rv1835c *628 aa* | Mb1866c *628 aa* | Hydrolase | Intermediary metabolism |
| 0063 (MT1980 *165 aa*) | Rv1928c *255 aa* | Mb1963c *255 aa* | Oxidoreductase | Intermediary metabolism |
| 0071 (MT2160.1 *56 aa* – MT2161 *954 aa*) | Rv2101 *1013 aa* | Mb2127 *1013 aa* | Helicase | Information pathway |
| 0073 (MT2312 *219 aa*) | Rv2252 *309 aa* | Mb2276 *309 aa* | Conserved hypothetical | Unknown |
| 0078 (MT2529 *345 aa*) | Rv2454c *373 aa* | Mb2481c *373 aa* | Oxidoreductase | Intermediary metabolism |
| 0079 (MT2762 *317 aa*) | Rv2688c *301 aa* | Mb2707c *301 aa* | ATP binding protein, ABC transporter | Cell wall, process |
| 0080 (MT2899 *350 aa*) | Rv2833c *436 aa* | Mb2857c *436 aa* | Sn-glycerol-3-phosphate-binding lipoprotein UgpB | Cell wall, process |
| 0087 (MT3177 *336 aa*) | Rv3093c *334 aa* | Mb3120c *334 aa* | Oxidoreductase | Intermediary metabolism |
| 0093 (MT3414 *294 aa*) | Rv3313c *365 aa* | Mb3342c *365 aa* | Adenosine deaminase | Intermediary metabolism |
| 0095 (MT3447 *544 aa*) | Rv3343c *2523 aa* | Mb3375c *1338 aa* | PPE family protein | PE/PPE |
| 0096 (MT3452 *916 aa* - MT3453 *2181 aa*) | Rv3347c *3157 aa* | Mb3380c *2096 aa* | PPE family protein | PE/PPE |
| 0097 (MT3453 *2181 aa* – MT3454 *204 aa*) | Rv3347c *3157 aa* | Mb2379c *1061 aa* | PPE family protein | PE/PPE |
| 0106 (MT3573.9 *68 aa* - MT3573.10 *35 aa* - MT3573.11 *224 aa*) | Rv1576c *473 aa* | Mb1602c *473 aa* | PhiRV1 phage protein | IS/phage |
| 0109 (MT3636 *140 aa*) | Rv3532 *406 aa* | Mb3562 *406 aa* | PPE family protein | PE/PPE |
| 0110 (MT3659 *75 aa*) | Rv3555c *289 aa* | Mb3585c *289 aa* | Conserved hypothetical | Unknown |
| 0119 (MT4000 *422 aa*) | Rv3885c *537 aa* | Mb3915c *537 aa* | Hypothetical | Unknown |
| 0120 (MT4001 *135 aa*) | Rv3886c *550 aa* | Mb3916c *550 aa* | Secreted alanine and proline rich protease MycP2 | Intermediary metabolism |

List of the ICDSs specific to *M. tuberculosis* CDC1551 (corresponding to full-length ORF in *M. tuberculosis* H37Rv and in *M. bovis* AF2122/97). The ICDS number, the affected ORF, the size of the predicted protein and its putative function are indicated. The size (in amino acid) of the corresponding predicted protein in the genome of *M. tuberculosis* H37Rv and *M. bovis* AF2122/97 is indicated.
