# Supplementary material for: Detecting the molecular scars of evolution in the Mycobacterium tuberculosis complex by analyzing interrupted coding sequences
Source: BMC Evol Biol. 2008 Mar 6;8:78. doi: 10.1186/1471-2148-8-78 (PMC2277376; doi:10.1186/1471-2148-8-78)
Supplement: Additional file 5 — Nucleotide sequence differences of the upstream region (200 bp) of the A- strain specific ICDS B- the full-length genes (control group). [file 1471-2148-8-78-S5.doc]

**Additional Table 5.**

A

| ***M. tuberculosis***  **H37Rv** | **ORF number** | **Putative function** | **Promotor in *M. bovis* AF2122/97** | **Promotor in *M. tuberculosis* CDC1551** |
| --- | --- | --- | --- | --- |
| 0006 | Rv0354c - Rv0355c | PPE family protein |  |  |
| 0008 | Rv0388c | PPE family protein |  |  |
| 0018 | Rv0781 - Rv0782 | Protease II |  |  |
| 0034 | Rv1180 - Rv1181 | Polyketide synthase Pks3/4 |  |  |
| **0044** | **Rv1783 - Rv1784** | **FtsK/SpoIIIE family protein** | **T-G at -20** |  |
| 0056 | Rv2250A -Rv2251 | Flavoprotein |  |  |
| 0057 | Rv2261c - Rv2262c | Conserved hypothetical |  |  |
| 0064 | Rv2879c - Rv2880c | Conserved hypothetical |  |  |
| 0081 | Rv3425 - Rv3426 | PPE family protein |  |  |
| 0082 | Rv3453 - Rv3454 | Conserved hypothetical |  |  |
| **0092** | **Rv3897c** | **Conserved hypothetical** | **T-C at -14** |  |
| 0096 | Rv3911 | Alternative RNA polymerase sigma factor SigM |  |  |
| ***M. tuberculosis***  **CDC1551** | **ORF number** | **Putative function** | **Promotor in *M. tuberculosis* H37Rv** | **Promotor in *M. bovis* AF2122/97** |
| 0001 | MT0028 - MT0029 | Hypothetical |  |  |
| 0002 | MT0032 | Hypothetical |  |  |
| 0006 | MT0170 | Oxidoreductase |  |  |
| 0008 | MT0279 | 5-oxo-L-prolinase |  |  |
| 0009 | MT0328 | Hypothetical |  |  |
| 0013 | MT0511 - MT0512 | Oxidoreductase |  |  |
| 0015 | MT0565 | Conserved hypothetical |  |  |
| **0023** | **MT0765 - MT0766** | **Hypothetical** |  | **A-G at -128** |
| **0024** | **MT0871** | **Cysteine synthase** |  | **C-T at -116** |
| 0025 | MT0872 | Conserved hypothetical |  |  |
| **0029** | **MT1004** | **PE_PGRS family protein** | **G-C at -50**  **G added at -151** | **G-C at -50** |
| 0033 | MT1099 | Hypothetical |  |  |
| 0038 | MT1157 | Hypothetical |  |  |
| **0043** | **MT1216 - MT1217** | **Hypothetical** | **G added at -93**  **G deleted at -40**  **C-T at -39** | **G added at -93** |
| 0045 | MT1408 - MT1409 | Hypothetical |  |  |
| 0047 | MT1469 | Carboxylesterase |  |  |
| **0049** | **MT1558** | **Hypothetical** |  | **Alignment with 16 nt** |
| 0050 | MT1578 | Polyketide synthase Pks5 |  |  |
| 0059 | MT1865 | Hypothetical |  |  |
| 0060 | MT1883 | Hydrolase |  |  |
| **0063** | **MT1980** | **Oxidoreductase** | **C added at -165** | **C added at -165** |
| 0071 | MT2160 - MT2161 | Helicase |  |  |
| **0073** | **MT2312** | **Conserved hypothetical** | **C-T at -14** |  |
| 0078 | MT2529 | Oxidoreductase |  |  |
| 0079 | MT2762 | ATP binding protein, ABC transporter |  |  |
| 0080 | MT2899 | Sn-glycerol-3-phosphate-binding lipoprotein UgpB |  |  |
| 0087 | MT3176 - MT3177 | Oxidoreductase |  |  |
| 0093 | MT3414 | Adinosine deaminase |  |  |
| **0095** | **MT3447** | **PPE family protein** | **Alignment with 136 nt** | **Alignment with 136 nt** |
| 0096 | MT3453 | PPE family protein |  |  |
| 0097 | MT3453 | PPE family protein |  |  |
| 0106 | MT3573.8/9/10/11 | PhiRV1 phage protein |  |  |
| 0109 | MT3636 | PPE family protein |  |  |
| 0110 | MT3659 - MT3660 | Conserved hypothetical |  |  |
| 0119 | MT4000 | Hypothetical |  |  |
| 0120 | MT4001 | Secreted alanine and proline rich protease MycP2 |  |  |
| ***M. bovis* AF2122/97** | **ORF number** | **Putative function** | **Promotor in *M. tuberculosis* H37Rv** | **Promotor in *M. tuberculosis* CDC1551** |
| 0001 | Mb0074-Mb0075 | Glutamine-transportABC transporter GlnQ |  |  |
| **0002** | **Mb0124c - Mb0125c** | **Elongation factor G** | **G-T at -100**  **G added at -41** | **T-C at -146**  **G-T at -100**  **G added at -41** |
| 0003 | Mb0139-1/2 | Epoxide hydrolase EphF |  |  |
| 0004 | Mb0139-2/3 | Epoxide hydrolase EphF |  |  |
| 0005 | Mb0139-3/4 | Epoxide hydrolase EphF |  |  |
| 0008 | Mb0228c | Aldehyde dehydroginase |  |  |
| 0012 | Mb0412 - Mb0413 | Polyketide synthase Pks6 |  |  |
| 0014 | Mb0563c - Mb0564c | Conserved hypothetical |  |  |
| 0015 | Mb0585 | Ribonucleoside-diphosphate reductase NdrZ |  |  |
| 0016 | Mb0590c | Oxidoreductase |  |  |
| **0017** | **Mb0609** | **MCE-family protein** | **C deleted at -137** | **C deleted at -137** |
| 0019 | Mb0646c - Mb0647c | Exonuclease V RecB |  |  |
| **0022** | **Mb0731 - Mb0732** | **Arylsulfatase AtsA** |  | **C-N at -161** |
| 0024 | Mb0819c | Oxidoreductase |  |  |
| 0026 | Mb0954c - Mb0955c | Serine/threonine protein kinase PknD |  |  |
| 0027 | Mb0957 - Mb0958 | phosphate-transport ABC transporter PstB |  |  |
| **0028** | **Mb1013 - Mb1014** | **Adhesion component transport ABC transporter** | **T-G at -57** | **T-G at -57** |
| 0029 | Mb1044c | Conserved lipoprotein LpqT |  |  |
| 0037 | Mb1290 - Mb1291 | Oxidoreductase |  |  |
| 0038 | Mb1345c - Mb1346c | Transposase |  |  |
| 0039 | Mb1350c - Mb1351c | Adapative response regulatory protein AlkA |  |  |
| 0040 | Mb1407 - Mb1408 | Glycolipid sulfotransferase |  |  |
| 0042 | Mb1540 - Mb1541 | Hypothetical |  |  |
| 0044 | Mb1746 | Conserved hypothetical |  |  |
| **0045** | **Mb1785c** | **Glycosyltransferase** | **Not found** |  |
| 0047 | Mb1791 | Conserved hypothetical |  |  |
| **0048** | **Mb1831c - Mb1832c** | **PE-PGRS family protein** | **G-A at -17** | **G-A at -17** |
| 0049 | Mb1838 - Mb1839 | PPE family protein |  |  |
| 0050 | Mb1875c | 6-phosphogluconate dehydrogenase Gnd1 |  |  |
| **0051** | **Mb1908- Mb1909** | **Conserved hypothetical** | **T-C at -40** | **T-C at -40** |
| 0057 | Mb2055c - Mb2056c | Conserved hypothetical |  |  |
| **0059** | **Mb2307c - Mb2308c** | **Conserved hypothetical** | **C-G at -93** | **G-A at -116**  **C-G at -93** |
| **0061** | **Mb2367 - Mb2368** | **Transmembrane transport protein MmpL9** | **G deleted at -132** | **G deleted at -132** |
| 0062 | Mb2595 - Mb2596 | Transglutaminase |  |  |
| 0063 | Mb2762c - Mb2763c | Conserved hypothetical |  |  |
| **0064** | **Mb2859c - Mb2860c** | **Sn-glycerol-3-phosphate-binding lipoprotein UgpA** | **A-G at -149** | **A-G at -149** |
| 0067 | Mb2982c | Glycosyltransferase |  |  |
| 0070 | Mb3201c - Mb3202c | Epoxide hydrolase MesT |  |  |
| **0073** | **Mb3507 - Mb3508** | **Conserved hypothetical** | **A-C at -132** | **A-C at -132** |
| 0074 | Mb3509c - Mb3510c | Conserved hypothetical |  |  |
| **0075** | **Mb3547c - Mb3548c** | **Cytochrome P450 monooxygenase1 Cyp142** |  | **Not found** |
| **0077** | **Mb3712c** | **Anti-anti-sigma factor RsfB** | **T-G at -20** | **T-G at -20** |
| **0080** | **Mb3827 - Mb3828** | **Transposase** |  | **Alignment with 110 nt** |
| 0081 | Mb3923c - Mb3924c | FtsK/SpoIIIE family protein |  |  |
| **0082** | **Mb3926c** | **Conserved hypothetical** | **A-G at -116** | **A-G at -116** |
| 0091 | Mb1200c - Mb1201c | PPE family protein |  |  |
| 0095 | Mb2314 | Thiosulfate sulfurtransferase SseB |  |  |
| **0105** | **Mb3507** | **Hypothetical** | **A-G at -1**  **A-C at -92** | **A-G at -1**  **A-C at -92** |
| **0106** | **Mb3507** | **Hypothetical** | **A-G at -1**  **A-C at -92** | **A-G at -1**  **A-C at -92** |
| 0110 | Mb3935c | ESAT-6 like protein EsxF |  |  |
| 0131 | Mb3436c | Aminotransferase |  |  |

B

| **ORF Number** | | | **Putative function** | **Promotor *M.* *tuberculosis* CDC1551** | **Promotor *M. bovis* AF2122/97** |
| --- | --- | --- | --- | --- | --- |
| ***M. tuberculosis* H37Rv** | ***M. tuberculosis* CDC1551** | ***M. bovis* AF2122/97** |
| Rv0004 | MT0004 | Mb0004 | Conserved hypothetical |  |  |
| Rv0036c | MT0041 | Mb0037c | Conserved hypothetical |  |  |
| Rv0088 | MT0096 | Mb0091 | Hypothetical |  |  |
| **Rv0148** | **MT0156** | **Mb0153** | **Short-chain type dehydrogenase/reductase** | **C-A at -148** |  |
| Rv0171 | MT0180 | Mb0177 | MCE-family protein Mce1C |  |  |
| Rv0190 | MT0200 | Mb0196 | Conserved hypothetical |  |  |
| **Rv0239** | **MT0253** | **Mb0245** | **Conserved hypothetical, antitoxin** |  | **C-T at -152** |
| Rv0267 | MT0280 | Mb0273 | Nitrite extrusion protein NarU |  |  |
| Rv0295c | MT0308 | Mb0303c | Conserved hypothetical |  |  |
| **Rv0317c** | **MT0332** | **Mb0325c** | **Glycerophosphoryl diester phosphodiesterase GlpQ2** |  | **T-C at -117** |
| Rv0321 | MT0336 | Mb0329 | Deoxycytidine triphosphate deaminase Dcd |  |  |
| Rv0371c | MT0386 | Mb0378c | Conserved hypothetical |  |  |
| **Rv0396** | **MT0406** | **Mb0402** | **Hypothetical** | **T-C at -171** | **T-C at -171** |
| Rv0463 | MT0479 | Mb0472 | Conserved membrane protein |  |  |
| **Rv0561c** | **MT0587** | **Mb0576c** | **Oxidoreductase** |  | **C-T at -180** |
| **Rv0604** | **MT0632** | **Mb0620** | **Conserved lipoprotein LpqO** |  | **C-G at -156** |
| Rv0635 | MT0664 | Mb0654 | Conserved hypothetical |  |  |
| Rv0702 | MT0729 | Mb0722 | 50S ribosomal protein L4 RplD |  |  |
| Rv0708 | MT0735 | Mb0728 | 50S ribosomal protein L16 RplP |  |  |
| Rv0789c | MT0814 | Mb0813c | Hypothetical |  |  |
| Rv0812 | MT0833 | Mb0835 | Amino acid aminotransferase |  |  |
| Rv0834c | MT0855 | Mb0857c | PE-PGRS family protein |  |  |
| **Rv0847** | **MT0870** | **Mb0870** | **Lipoprotein LpqS** |  | **A-G at -46** |
| Rv0887c | MT0910 | Mb0911c | Conserved hypothetical |  |  |
| Rv0914c | MT0939 | Mb0938c | Lipid carrier protein or keto acyl-coA thiolase |  |  |
| Rv0940c | MT0967 | Mb0965c | Oxidoreductase |  |  |
| Rv0954 | MT0981 | Mb0979 | Conserved transmembrane protein |  |  |
| Rv0968 | MT0996 | Mb0993 | Conserved hypothetical |  |  |
| Rv1061 | MT1091 | Mb1090 | Conserved hypothetical |  |  |
| Rv1078 | MT1109 | Mb1107 | Proline-rich antigen homolog Pra |  |  |
| **Rv1104** | **MT1136** | **Mb1134** | **Para-nitrobinzyl esterase** | **A added at -37** |  |
| Rv1242 | MT1280 | Mb1274 | Conserved hypothetical with PIN domain |  |  |
| **Rv1261c** | **MT1299** | **Mb1292c** | **Conserved hypothetical** |  | **G-C at -129** |
| Rv1311 | MT1351 | Mb1343 | ATP synthase epsilon chain AtpC |  |  |
| Rv1392 | MT1437 | Mb1427 | S-adinosylmethionine synthetase MetK |  |  |
| Rv1411c | MT1455 | Mb1446c | Conserved lipoprotein LprG |  |  |
| Rv1470 | MT1516 | Mb1505 | Thioredoxin TrxA |  |  |
| Rv1524 | MT1575 | Mb1551 | Probable glycosyltransferase |  |  |
| **Rv1571** | **MT1622** | **Mb1598** | **Conserved hypothetical** | **T-C at -108** | **T-C at -108** |
| Rv1592c | MT1628 | Mb1618c | Conserved hypothetical |  |  |
| Rv1615 | MT1651 | Mb1641 | Hypothetical membrane protein |  |  |
| **Rv1623c** | **MT1659** | **Mb1649c** | **Cytochrome D ubiquinol oxidase cydA** |  | **T-C at -167** |
| Rv1662 | MT1702 | Mb1690 | Polyketide synthase Pks8 |  |  |
| Rv1695 | MT1734 | Mb1721 | Inorganic polyphosphate/ATP-NAD kinase PpnK |  |  |
| Rv1747 | MT1789 | Mb1776 | ATP-binding protein, ABC transporter |  |  |
| Rv1749c | MT1792 | Mb1778c | Integral membrane protein |  |  |
| Rv1892 | MT1942 | Mb1925 | Membrane protein |  |  |
| Rv1898 | MT1949 | Mb1933 | Conserved hypothetical |  |  |
| Rv1952 | MT2002 | Mb1987 | Conserved hypothetical, antitoxin |  |  |
| **Rv2115c** | **MT2175** | **Mb2139c** | **ATPase** |  | **A-G at -135** |
| Rv2145c | MT2204 | Mb2169c | Conserved hypothetical WAG31 |  |  |
| Rv2201 | MT2257 | Mb2224 | Asparagine synthetase AsnB |  |  |
| Rv2249c | MT2309 | Mb2273c | Glycerol-3-phosphate dehydroginase GlpD1 |  |  |
| **Rv2287** | **MT2345** | **Mb2309** | **Conserved integral membrane transport protein YjcE** | **C-T at -16** | **C-G at -41** |
| Rv2324 | MT2386 | Mb2351 | Transcriptional regulatory protein |  |  |
| Rv2362c | MT2431 | Mb2383c | DNA repair protein RecO |  |  |
| Rv2364c | MT2433 | Mb2385c | GTP-binding protein Era |  |  |
| Rv2474c | MT2549 | Mb2501c | Conserved hypothetical |  |  |
| Rv2494 | MT2569 | Mb2522 | Conserved hypothetical with PIN domain |  |  |
| Rv2522c | MT2598 | Mb2551c | Conserved hypothetical |  |  |
| Rv2560 | MT2637 | Mb2590 | Proline and glycine rich transmembrane protein |  |  |
| **Rv2562** | **MT2638** | **Mb2591** | **Conserved hypothetical** | **Alignment with 192 nt** | **Alignment with 192 nt** |
| **Rv2618** | **MT2693** | **Mb2651** | **Conserved hypothetical** |  | **T-C at -105** |
| Rv2688c | MT2762 | Mb2707c | ATP-binding protein, ABC transporter |  |  |
| Rv2697c | MT2771 | Mb2716c | Deoxyuridine 5'-triphosphate nucleotidohydrolase Dut |  |  |
| Rv2745c | MT2816 | Mb2766c | Transcriptional regulatory protein |  |  |
| Rv2758c | MT2828 | Mb2779c | Conserved hypothetical protein, Antitoxin |  |  |
| Rv2801c | MT2869 | Mb2824c | Conserved hypothetical |  |  |
| Rv2896c | MT2964 | Mb2920c | Conserved hypothetical |  |  |
| Rv2909c | MT2977 | Mb2933c | 30S ribosomal protein S16 RpsP |  |  |
| Rv2967c | MT3045 | Mb2991c | Pyruvate carboxylase Pca |  |  |
| Rv2977c | MT3055 | Mb3001c | Thiamine-monophosphate kinase ThiL |  |  |
| Rv3028c | MT3112 | Mb3054c | Electron transfer flavoprotein (alpha-subunit) FixB |  |  |
| **Rv3045** | **MT3130** | **Mb3071** | **NADP-dependent alcohol dehydrogenase AdhC** |  | **G-A at -27** |
| Rv3086 | MT3171 | Mb3113 | Zinc-type alcohol dehydrogenase AdhD |  |  |
| Rv3142c | MT3229 | Mb3166c | Hypothetical |  |  |
| Rv3191c | MT3281 | Mb3213c | Transposase |  |  |
| Rv3228 | MT3325 | Mb3257 | Conserved Hypothetical |  |  |
| Rv3246c | MT3344 | Mb3274c | Two component sensory transduction transcriptional regulatory protein MtrA |  |  |
| Rv3309c | MT3408 | Mb3337c | Uracil phosphoribosyltransferase Upp |  |  |
| Rv3332 | MT3435 | Mb3365 | N-acetylglucosamine-6-phosphate deacetylase NagA |  |  |
| Rv3416 | MT3525 | Mb3450 | Transcriptional regulatory protein WhiB3 |  |  |
| **Rv3454** | **MT3561** | **Mb3483** | **Conserved integral membrane protein** | **T deleted at -99** | **T deleted at -99** |
| Rv3494c | MT3598 | Mb3524c | MCE-family protein Mce4F |  |  |
| Rv3531c | MT3634 | Mb3561c | Hypothetical |  |  |
| Rv3558 | MT3663 | Mb3588 | PPE family protein |  |  |
| Rv3568c | MT3673 | Mb3599c | Biphenyl-2,3-diol 1,2-dioxygenase BphC |  |  |
| Rv3605c | MT3710 | Mb3635c | Conserved secreted protein |  |  |
| Rv3676 | MT3777 | Mb3700 | Transcriptional regulatory protein |  |  |
| **Rv3713** | **MT3816** | **Mb3740** | **Cobyric acid synthase CobQ2** |  | **A-C at -194** |
| Rv3806c | MT3913 | Mb3836c | Conserved integral membrane protein |  |  |
| Rv3813c | MT3922 | Mb3843c | Conserved hypothetical protein |  |  |
| Rv3820c | MT3928 | Mb3850c | Polyketide synthase associated protein PapA2 |  |  |
| Rv3846 | MT3960 | Mb3876 | Superoxide dismutase SodA |  |  |
| Rv3853 | MT3968 | Mb3883 | Regulator of RNase E activity A RraA |  |  |
| **Rv3887c** | **MT4002** | **Mb3917c** | **Conserved transmembrane protein** |  | **Alignment with 16 nt** |
| Rv3890c | MT4005 | Mb3919c | ESAT-6 like protein EsxC |  |  |
| **Rv3898c** | **MT4014** | **Mb3927c** | **Conserved hypothetical** |  | **C-G at -22** |
| Rv3902c | MT4020 | Mb3932c | Hypothetical |  |  |

Nucleotide sequence differences of the upstream region (200 bp) of the **A-** strain specific ICDS **B-** the full-length genes (control group). The location of the nucleotide variation compared to translation initiation site is indicated.
